# Acceptability, Feasibility, and Effectiveness of Immersive Virtual Technologies to Promote Exercise in Older Adults: A Systematic Review and Meta-Analysis

**DOI:** 10.3390/s23052506

**Published:** 2023-02-24

**Authors:** Benjamin Doré, Alex Gaudreault, Gauthier Everard, Johannes C. Ayena, Ahmad Abboud, Nicolas Robitaille, Charles Sebiyo Batcho

**Affiliations:** 1 Department of Rehabilitation, Faculty of Medicine, Laval University, Quebec, QC G1V 0A6, Canada; 2 Center for Interdisciplinary Research in Rehabilitation and Social Integration (Cirris), Centre Intégré Universitaire de Santé et de Services Sociaux de la Capitale Nationale (CIUSSS-CN), Quebec, QC G1M 2S8, Canada; 3 Alborea, Quebec, QC G1V 0A6, Canada

**Keywords:** immersive technology, virtual reality, augmented reality, elderly, exercise

## Abstract

Context: This review aimed to synthesize the literature on the acceptability, feasibility, and effectiveness of immersive virtual technologies to promote physical exercise in older people. Method: We performed a literature review, based on four databases (PubMed, CINAHL, Embase, and Scopus; last search: 30 January 2023). Eligible studies had to use immersive technology with participants aged 60 years and over. The results regarding acceptability, feasibility, and effectiveness of immersive technology-based interventions in older people were extracted. The standardized mean differences were then computed using a random model effect. Results: In total, 54 relevant studies (1853 participants) were identified through search strategies. Concerning the acceptability, most participants reported a pleasant experience and a desire to use the technology again. The average increase in the pre/post Simulator Sickness Questionnaire score was 0.43 in healthy subjects and 3.23 in subjects with neurological disorders, demonstrating this technology’s feasibility. Regarding the effectiveness, our meta-analysis showed a positive effect of the use of virtual reality technology on balance (SMD = 1.05; 95% CI: 0.75–1.36; *p* < 0.001) and gait outcomes (SMD = 0.7; 95% CI: 0.14–0.80; *p* < 0.001). However, these results suffered from inconsistency and the number of trials dealing with these outcomes remains low, calling for further studies. Conclusions: Virtual reality seems to be well accepted by older people and its use with this population is feasible. However, more studies are needed to conclude its effectiveness in promoting exercise in older people.

## 1. Introduction

According to the World Health Organization, the number of people aged 60 years and over will reach 2 billion by 2050, while those aged 80 years and above are expected to grow from 125 million (in 2018) to 434 million in 2050 [1]. This accelerated aging currently observed in most industrialized countries is causing an increase in the prevalence of people with functional limitations related to mobility and fall risks. Indeed, aging results in a progressive decline of different body functions, leading to a higher risk of morbidity [2] and recurrent balance and walking disorders. Over the age of 65 years, more than a third of people fall at least once a year [3] as gait and balance disorders increase with age [4]. Given their prevalence and the physical, physiological, and psychological impact in older people, falls are a significant concern for health systems. Falls are predictors of decreased social participation [5]. It is therefore critical to find effective avenues for helping older people to prevent falls and rehabilitate balance disorders in order to maintain their independence in daily activities.

Many studies, as summarized in [6,7], have shown that rehabilitation can play a fundamental role in reducing the consequences related to balance disorders while improving the efficiency of the health system [8]. Among other rehabilitation interventions, physiotherapy, with interventions aimed at improving balance and strength, offers promising features in the prevention of falls in populations at risk [9]. However, while studies have shown that conventional exercises could reduce the risk of falling by 21% in older people, this still requires a certain amount of practice (at least three hours per week) to be effective. Reducing the risk of falling through conventional physiotherapy therefore demands time, availability, great treatment adherence, and frequent visits to the hospital or rehabilitation center [10]. Recent technological developments such as virtual and augmented reality technologies might be a solution to these needs. This technology provides interesting potential to increase treatment intensity and deliver remote or unsupervised rehabilitation for patients who do not have access to healthcare systems, for economic or geographical reasons.

Virtual reality (VR) is often defined as immersive or non-immersive according to the devices used to submerse users’ senses. In immersive VR, the immersion is created through the use of a head-mounted display or a cave automatic virtual environment (CAVE). VR must also be distinguished from mixed reality systems such as augmented reality (AR), where real-world elements are being included into the virtual environment [11]. In this review, immersive VR and AR were categorized as immersive technologies.

Immersive virtual technologies have emerged as effective tools to perform exercises aimed at improving balance and strength in the community-dwelling adults [12,13]. These technologies also appear beneficial for promoting engagement and motivation in physical activity interventions. VR and AR can be used at different stages of the physical rehabilitation process, i.e., for assessment, treatment, or research purposes [14]. Such technologies offer interesting possibilities for neurorehabilitation using tridimensional environments, multisensorial stimulations, and precise measures of kinematics [15]. As an example, these devices can be used as a relevant means to deliver interventions for improving walking in a Parkinson’s disease (PD) population [16], as well as to assess the displacement of the center of gravity and balance functions in both healthy older people and people with disability [17]. Immersive VR and AR can also be used to establish and tailor interventions according to the severity of gait and balance issues. Furthermore, as shown by Canning et al. [18], such technology offers potential to better understand the physiological mechanisms responsible for neurological diseases and to measure indicators of fall risk in the older people [19].

Although immersive technologies were found to be effective in numerous areas, their integration into clinical practice remains a challenge [20], with unknown evidence regarding their feasibility, acceptability, and effectiveness in older people. To the best of our knowledge, no systematic review has investigated all of these three main aspects through a single summarized literature review. In this paper, we, therefore, propose a new systematic review aiming to summarize the evidence on the feasibility and acceptability, and to evaluate the effectiveness of VR and AR in older people.

## 2. Materials and Methods

### 2.1. Search Strategy

This review has been performed in accordance with the Preferred Reporting Items for Systematic Reviews and Meta-Analyses (PRISMA) guidelines [21]. The search strategy was mainly directed toward finding published articles using four wide databases (MEDLINE via the PubMed platform, CINAHL Plus with Full Text via the EBSCOhost platform, Embase, and Scopus). Our search strategy was based on a mixture of indexed and free vocabulary keywords (Appendix A).

### 2.2. Eligibility Criteria

Studies were included if they reported results (1) addressing acceptability, feasibility, and/or effectiveness; (2) of immersive VR or AR technology in the physical therapy or rehabilitation context; (3) on adults with a mean age of 60 years or older (as defined by the United Nations); and (4) that were published in English or French, with no limit on the date. Systematic reviews with or without meta-analysis, reviews, conference or congress papers, and case report studies were excluded. Pre-post interventional studies assessing the effectiveness of immersive VR or AR in older adults and providing sufficient data to analyze changes in outcomes were included in the meta-analysis.

### 2.3. Study Selection

References retrieved from MEDLINE, CINAHL, Embase, and Scopus were exported into the Covidence systematic review software (Veritas Health Innovation, Melbourne, Australia. Available at www.covidence.org) [22]. In addition, two independent reviewers carefully reviewed the references list of relevant systematic reviews and meta-analyses to further extend the identification of potential articles according to the inclusion and exclusion criteria. The study selection was first carried out separately by two independent reviewers, with respect to the eligibility criteria described above. A consensus meeting was organized to resolve any discrepancy. This happened when reviewers differed in their respective decisions, or if one of them had doubts about the potential inclusion of a study. If the disagreement persisted, a third independent reviewer, blinded to the selections of the first two reviewers, was invited to screen and resolve the issue as a final decision.

### 2.4. Data Extraction

Two independent reviewers extracted the data from the included studies. Each reviewer had half of the articles selected to read. All relevant data were combined in a single Excel table (Microsoft 365). For each study, the following information was retrieved: the date of publication, the country in which the study took place, the population and its main characteristics (type of population, mean age, time since diagnosis of the pathology, degree of impairment if applicable, sample size, sex distribution), the experimentation performed (type of experimentation, duration of exposure in immersive environment, presence or absence of supervision, brand of immersive headset used if applicable), and the results and assessment methods for the three targeted outcomes (acceptability, feasibility, and effectiveness), as well as the authors’ conclusions.

### 2.5. Methodological Quality Assessment of the Selected Studies

Following the selection and the data extraction, each reviewer assessed the methodological quality of the randomized control trials using the PEDro scale [23]. This 11-item scale, containing up to 10 scoring criteria, was applied to determine the quality of each study’s methodology. However, in our review, criteria 5 and 6, related to the blinding of all subjects and blinding of all therapists who administered the intervention, respectively, were removed since it is impossible for subjects and therapists to be blinded in studies using such technologies. Therefore, the highest possible score for an article was 8, given that the first criteria was not designed to be scored [24].

Afterward, the score of each study was interpreted as suggested by Foley et al. [24], in which a score of 9 or 10 indicates an excellent methodological quality, a score of 6 to 8 means a good methodological quality, a score of 4 or 5 is considered as an acceptable methodological quality, and a score of <4 indicates poor methodological quality. As presented in Cashin et al. [25], this scoring method has demonstrated not only a moderate to excellent inter-rater reliability for clinical trials related to physiotherapy interventions but also a good convergent validity.

Regarding the non-randomized experimental studies, the National Institute of Health Quality Assessment Tool was used to assess their methodological quality, whereas for qualitative studies, the grid of the Centre for Evidence-Based Medicine (CEBM) for Critical Appraisal of Qualitative Studies [26] was used. This tool allows for the evaluation of the reliability, importance, and applicability of the reported clinical evidence. Finally, the evidence levels of the studies dealing with the effectiveness of the immersive technologies were determined using the Jovell and Navarro-Rubio scale [27]. In this scale, the study design is specified as one of 9 levels, in descending orders of strength (see Table 1 in [28]).

### 2.6. Statistical Analysis

Meta-analyses were considered when at least four studies provided quantitative measures of effect for the same outcome. The changes induced by VR and AR were computed from the included studies. For each relevant outcome, the following information was introduced into the RevMan 5.3 software: pre- and post-intervention mean scores ± standard deviation and the total number of participants. This enabled us to generate forest plots, underlining the treatment effectiveness. When different scales were used for one outcome, the standardized mean difference (SMD) and 95% confidence interval were calculated for each study. The magnitude of the effect was interpreted according to Cohen’s guidelines: small for SMD ≤ 0.5, medium for 0.5 < SMD ≤ 0.8, and large for SMD > 0.8 [29]. The I^2^ statistical test was also considered to estimate results’ heterogeneity. As suggested by the Cochrane Handbook, heterogeneity was defined as non-significant for I^2^ < 30%, moderate for 30% ≤ I^2^ < 50%, substantial for 50% ≤ I^2^ < 75%, and considerable for I^2^ ≥ 75%. In case of heterogeneity, a random effect model was always considered. Outlier study removal was always motivated by a sensitivity analysis. Subgroup analyses were considered to assess the influence of time (studies published after 2020 vs. before 2020), the type of device (AR vs. VR), and the participants’ health status (healthy older adults vs. older adults with any pathology) on immersive technologies effectiveness when at least 10 studies were included in the analysis.

The strength of the body of evidence was evaluated according to the GRADE approach. The certainty of the evidence was consequently established depending on the risk of bias of the included studies, the number of participants, the statistical heterogeneity, the effect size, and the design of the studies.

## 3. Results

**Figure 1:**
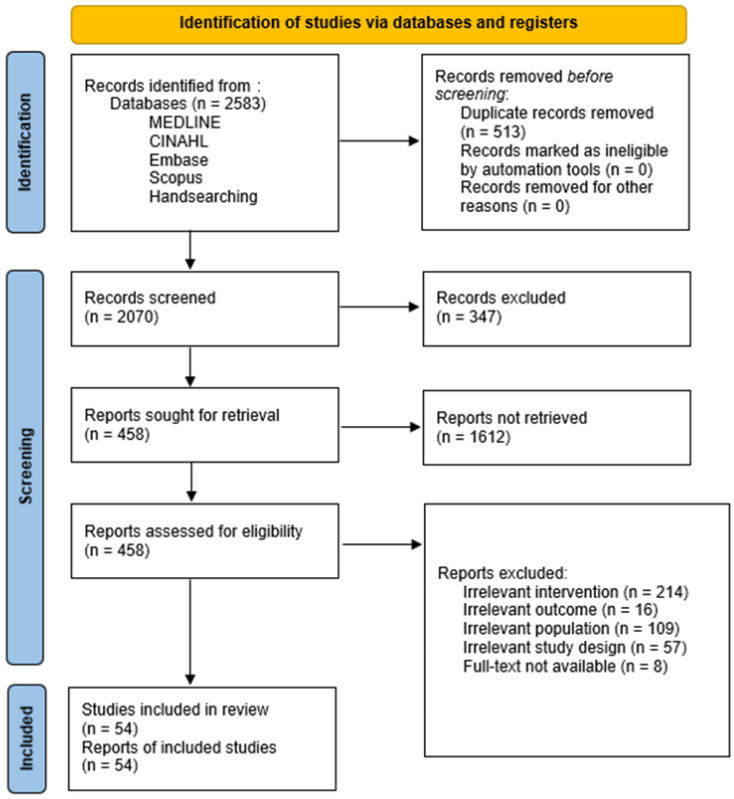
Flow chart diagram of included studies.

The electronic search strategy in the MEDLINE, CINAHL, Embase, and Scopus databases yielded 2542 records. Handsearching led to 41 additional articles (Figure 1). As a result, a total of 2583 articles were exported into the Covidence software [22]. After removing the duplicates, 2070 titles and abstracts were screened. A total of 54 different studies (1853 participants) were finally selected. These studies were issued from 23 different countries (Table 1). The years of publication ranged from 2006 to 2022. In total, 91% of the included studies were published after 2015 and 67% were published in 2020 or later. In the next subsections, we report the most important findings.

### 3.1. Characteristics of the Experiment Designed in the Selected Studies

As shown in Table 1, 43 of the 54 studies used a VR headset. In total, 19 studies [31,32,35,37,38,42,47,49,54,59,60,62,63,64,65,66,67,68] had used the HTC Vive, 8 studies [16,34,39,41,46,52,58,61,71] used the Oculus Rift, 3 studies [51,55,56] used the Glasstron LDI-100B, 3 studies used the Oculus Quest [36,43,69], and 3 studies [30,33,45] used the Samsung Gear VR. The remaining studies used the following headsets: Revelation 3D VR Headset with a Lumia 930 phone [53], University of Ulster’s Virtual Reality Rehabilitation (UUVRR) System [40], Valve Index [48], VR GLASS [70], and Balance Rehabilitation Unit (BRU) [57]. Jung et al. [44] did not mention the type of VR headset used in their study. The following AR headsets were also used in different studies: AIRO II [72], Glasstron PLM-5700 [76], Laster WAVƎ [73], Microsoft Kinect [74,78], NEURO RAR [79], Portable Exergame Platform for Elderly (PEPE) [75], Microsoft HoloLens [67], UNICARE HEALTH [77], and i-visor FX601 [81].

Table 1 also reports the different types of populations groups, as well as the average age, the time since diagnosis, and the severity of illness when available in the selected paper. In total, 1 study [30] (using VR technology) included subjects with mild to moderate dementia, 6 studies included participants with Parkinson’s disease (5 VR and 1 AR [72]), 29 articles (22 VR, 7 AR, and 1 CAVE [82]) included healthy older people, 7 studies (3 VR and 4 AR) included people with stroke, 3 VR studies included patients with pain affecting their daily activities, 2 VR studies included patients with vestibular impairments, 2 VR studies included subjects at risk of falling, 3 VR studies included patients with cognitive impairments, 1 VR study [55] included a patient with a total knee replacement, 1 VR study [71] included a patient suffering from functional incapacities, 1 VR study [62] included a patient with hypertension, and 1 VR study [52] included a patient with a distal radius fracture.

In total, 10 studies (9 VR and 1 CAVE [82]) exposed their participants for no more than 15 min per session. In 18 studies using an immersive technology, the participants were exposed to a maximum of 30 min per session, whereas 10 studies (8 VR and 2 AR) exposed their participants to more than 30 min per session. Furthermore, 13 studies (9 VR and 5 AR) did not mention the exposure duration. As reported in Table 1, 30 studies (24 VR and 6 AR) exposed participants to several VR sessions. In 45 studies (36 VR, 10 AR, and 1 CAVE [82]), the participants were supervised during their experimentation. Finally, 9 studies (7 VR and 2 AR) did not report whether supervision was provided to participants while exposed to the virtual environment.

### 3.2. Methodological Quality Assessment

Table 2, Table 3 and Table 4 present the methodological quality assessment of the different studies included in this review. According to the PEDro scale (Table 2), 16 studies (14 VR and 2 AR) showed good quality and 14 studies (10 VR and 4 AR) showed acceptable quality. Regarding the non-experimental studies, the results are presented in Table 3. Based on the CEBM scale (Table 4), four qualitative studies (three VR and one CAVE) could be classified as of good methodological quality. However, the small sample sizes of these studies limit the generalizability of their respective findings.

### 3.3. Findings on the Acceptability, Feasibility, and Effectiveness

#### 3.3.1. Acceptability

Twenty-one articles (Table 5) have addressed the acceptability of VR [16,30,32,33,36,39,41,42,48,49,50,52,57,62,64,66,67,80,82]). Syed-Abdul et al. [64] indicated that the headset (HTC Vive) was comfortable for the participants. Appel et al. [30] and Benham et al. [32] indicated that the participants found the immersive VR experience enjoyable (via a home questionnaire showing a high satisfaction rate). Brown [33], De Keersmaecker et al. [16], and Syed-Abdul et al. [64] also reported that their participants enjoyed the experience. In Appel et al. [30] and Brown [33], the participants reported that they would be willing to repeat the experience in the future if they had the opportunity. Benham et al. [32] showed that older people were very keen to try this new technology and Phu et al. [57] observed a similar rate of treatment adherence between the conventional exercise group and the immersive VR group, contrary to Cikajlo and Peterlin Potisk [39] and Syed-Abdul et al. [64] who reported a higher motivation towards the treatment in the VR groups compared to the conventional treatment groups.

Janeh et al. [42] highlighted a moderate level of immersion and low fear of physical contact with the real environment during immersion. Syed-Abdul et al. [64] concluded that older people consider using a technology based on its ease and usefulness. Indeed, the enjoyment obtained during the experiences, as well as the perception of their participants, provided positive attitudes concerning the use of this new technology. No study has evaluated the acceptability of AR-based interventions and only one study addressed the acceptability of the CAVE system. Pedroli et al. [82] found that their participants were highly engaged when immersed in the CAVE environment. Accordingly, it appears that most participants reported that they forgot the training context, which could be responsible for their increasing implication in rehabilitation.

#### 3.3.2. Feasibility

Twelve studies [16,35,36,37,38,41,46,48,53,56,61,64] used the Simulator Sickness Questionnaire (SSQ) [83] to assess the feasibility of immersive technology (Table 6). This questionnaire was administered before and after VR exposure. Saldana et al. [61] administered the SSQ questionnaire over two sessions and observed a decrease in the total score among the group using VR at the second assessment session. Indeed, the difference before and after exposure to the technology were −1.38 ± 2.29 at the first session and −0.25 ± 1.91 at the second session, indicating that fewer symptoms were present at the second visit. However, other studies [16,46,53,55] showed that, for a healthy population, the score averaged from 7.78 (2.39–16.45) before exposure to VR to 10.23 (1.36–15.21) after exposure to the technology, which, compared to populations with various health conditions, indicates an increase in the symptoms of discomfort related to the simulation. Across the papers addressing feasibility, while other works reported a decrease in the experienced side effects [42], opposite trends (an increase after immersion) were observed in [46].

Appel et al. [30] carried out VR testing in which the data were collected during pre/post-intervention. They concluded that there were no negative side effects to using the VR technology in the neurologically impaired population. Most of the participants had positive feedback and felt more relaxed, with a decrease in anxiety (1.96 ± 1.55 to 1.81 ± 1.51), stress (1.94 ± 1.5 to 1.86 ± 1.55), tension (1.48 ± 1.11 to 1.34 ± 0.83), and feeling upset (1.82 ± 1.25 to 1.42 ± 1.12).

With a home-built questionnaire evaluating the usability and engagement in AR, Bank et al. [72] reported a mean score of 69.3 ± 13.7 out of 100 for the usability section, indicating that the use of such technology was possible, and a mean score of 3.8 ± 0.5 out of 5 for engagement, which could be considered as moderate engagement. The ease of use and realism in manipulating objects are elements that may affect this sense of engagement. They concluded that AR is well tolerated and participants’ augmented experiences were close to real experiences. Crosbie et al. [40] assessed the physical demands of using VR with the Borg scale [84], ranging from 0 to 10. The perceived exertion score in the virtual environment was 5.6 ± 2.22 in the stroke group and 1.6 ± 1.24 in the healthy group, indicating that performing tasks in the virtual environment appeared to be more difficult for people with a stroke than healthy people. However, both groups reported favorable experiences with VR, even though the stroke group faced greater physical demands with completing the same tasks.

#### 3.3.3. Effectiveness

##### Meta-Analysis

**Figure 2:**
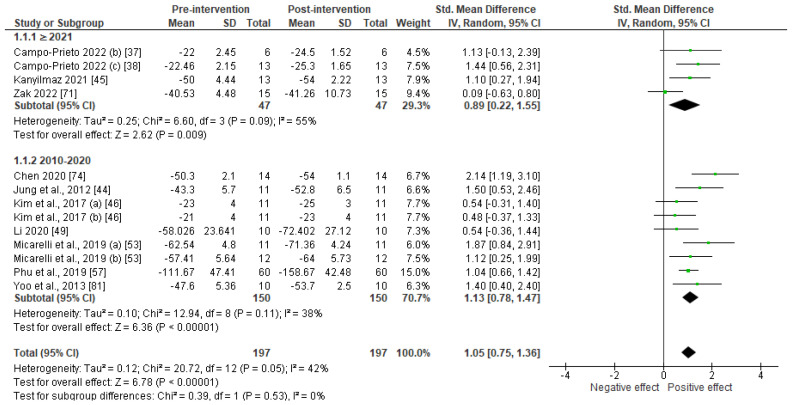
Forest plot of effects of intervention using immersive technologies on balance.

There was sufficient data (n ≥ 4) to quantify the effect of VR and AR on older adults’ balance and gait functions. Thirteen studies used instrumental measures to assess balance outcomes. These were the ABC scale, the Mini-BESTest, the Berg Balance Scale, the Tinetti Balance Test, the One-Leg Standing Balance Test, and the limits of stability (a posturography index). As underlined in Figure 2, AR and VR led to significant improvements in the balance function (SMD = 1.05; 95% CI: 0.75–1.36; *p* < 0.001) with a large magnitude of effect (SMD > 0.8). However, the heterogeneity between the studies was found to be moderate (I^2^ = 42%). According to the GRADE approach, owing to the limited number of studies and sample size, the potential risk of bias, and the significant heterogeneity of these results, the strength of the body of evidence was decreased by two and therefore considered as low.

Subgroup analyses revealed that the effect of immersive technologies on balance outcomes was not influenced by the years (*p* = 0.53). The studies published before 2021 (SMD = 0.89; 95% CI: 0.22–1.55; *p* = 0.009) led to similar balance benefits as the studies published between 2010 and 2020 (SMD = 1.13; 95% CI: 0.78–1.47; *p* < 0.001).

**Figure 3:**
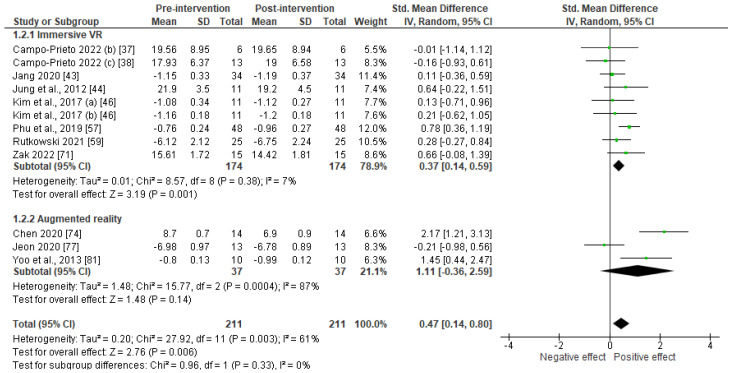
Forest plot of effects of intervention using immersive technologies on gait speed.

Fourteen studies used instrumental measures of gait outcomes. These were the gait speed, six-minute walk test, and Timed Up and Go test. As underlined in Figure 3, immersive technologies were found to significantly improve the outcome (SMD = 0.47; 95% CI: 0.14–0.80; *p* < 0.006). The effect size was considered as moderate (0.2 < SMD < 0.8) and the heterogeneity between the studies was found to be substantial (I^2^ = 61%). Given the low number of included studies, the potential risk of bias, the moderate effect size, and the substantial heterogeneity, the certainty of evidence was considered as very low.

Subgroup analyses revealed that immersive VR led to significant gait speed improvements (SMD = 0.37; 95% CI: 0.14–0.59; *p* = 0.001), whereas AR did not significantly enhance the gait outcomes (SMD = 1.11; 95% CI = −0.36–2.59; *p* = 0.14). However, as underlined by Appendix B, the effect of these technologies substantially differed according to the pathology.

##### Studies Results

Kim et al. [46] used the Mini-BESTest to assess the balance in healthy participants and people with Parkinson’s disease. For the healthy population, the pre-intervention balance score of 23 ± 4 changed to 25 ± 3 after experimentation with VR, while in participants with Parkinson’s, the score increased from 21 ± 4 to 23 ± 4, with the change being statistically significant in each group (F (2,30) = 5.33, *p* < 0.05). They also reported a gait speed improvement after VR exposure. The participants walked significantly faster after exposure, from 1.08 ± 0.34 m/s to 1.12 ± 0.27 m/s and from 1.16 ± 0.18 m/s to 1.20 ± 0.18 m/s, respectively, for healthy adults and people with Parkinson’s disease. Yoo et al. [81] also reported that AR contributed to a positive change in gait parameters, balance, and fall risk in older people after AR exposure.

Phu et al. [81] also investigated the gait speed in relation to the use of the BRU. This VR platform resulted in a significant 12% improvement in walking speed. The authors also observed a significant decrease in the risk of falling after the use of BRU. Indeed, the Falls Efficacy Scale–International (FES-I) post-exposure score decreased by 11.3 points, while the Five Times Sit-to-Stand (FTSTS) showed a significant decrease of 26.69% in the time required to complete the five repetitions. These two results led to the conclusion that BRU might be effective at reducing the risk of falls in older people. Two other studies [44,53] used the Activities-specific Balance Confidence scale (ABC) to quantify balance in people with vestibular impairment [53] and stroke [44] and observed a significant improvement in the performance with the ABC mean scores changing from 62.54 ± 4.8 to 71.36 ± 4.24 [53]. Jung et al. [44] reported an improvement of 9.5% ± 6.0%. It appears that VR training can improve the perception of balance in people with health problems. The researchers also used the Timed Up and Go (TUG) test to evaluate the potential effects of VR. They reported a mean decrease of 2.7 ± 1.9 sec in the time to complete the test after exposure to VR, showing an improvement in gait balance.

Janeh et al. [42] used the GAIT-Rite system to analyze different walking parameters before and after the use of a VR device. The length of the shortest step increased from 58.34 ± 8.27 cm to 60.45 ± 8.16 cm after exposure, while the walking symmetry varied from 1.05 ± 0.04% to 1.01 ± 0.06%. In this study, the cadence before exposure to VR was 102.81 ± 8.19 steps/min and this changed to 97.41 ± 9.9 steps/min after exposure to VR. The cadence parameter was also used by Yoo et al. [81] to document the effects of AR. The cadence before exposure to AR was 100.79 ± 9.92 steps/min and this increased to 116.73 ± 8.81 steps/min after exposure, indicating an increase in the walking cadence. It can therefore be suggested that, unlike the immersive VR used by Janeh et al. [42], AR leads to an increase in the walking cadence. Yoo et al.’s study [81] also found a significant increase in the Berg Balance Scale scores (47.60 ± 5.36 before and 53.50 ± 2.30 after exposure to AR).

Benham et al. [32] used VR to address pain. The Numeric Pain Rating Scale (NPRS) score showed a significant decrease, with pain scores changing from 3.5 ± 1.73 to 0.9 ± 1.62 after exposure to VR. Their outcomes also included the World Health Organization Quality of Life Scale Brief Version (WHOQOL-BREF), where no effect was reported. In conclusion, we can note that there is a significant improvement in pain via the distraction provided by VR.

Furthermore, as presented in Table 7, some papers (VR [39,57], AR [72,76]) focused on the upper limbs. Phu et al. [57] investigated the grip strength and found that there was a significant improvement in the grip strength in the immersive VR users. Indeed, the BRU group reported a significant increase (*p* = 0.027) of 6.82% over the initial score [57]. Fischer et al. [76] used AR coupled with a pneumatic orthosis for the upper limb. This study reported a significant increase in the task performance on the Wolf Motor Function Test (WMFT), which was illustrated by a 12.9-point decrease (*p* = 0.02). However, they did not report a significant change in the biomechanical measures of hand or grip strength (*p* > 0.20) but reported that the AR would allow faster transitions between tasks and more opportunities to practice gripping objects that would not be available in the conventional clinical environment.

Lastly, Kanyilmaz et al. have assessed the effect of immersive VR on older adults suffering from dizziness [45]. The results of this work showed that the combination of immersive VR and vestibular rehabilitation offers greater vertigo improvements at 6 months post-intervention than vestibular rehabilitation alone.

## 4. Discussion

This review summarized what is currently known about the use of immersive VR and AR technologies in older people. The following subsections discuss the results regarding the main research purpose, such as the acceptability, the feasibility, and the effectiveness of VR. We also highlight the limitations of the present study.

### 4.1. Acceptability

Our review identified 21 articles addressing the acceptability of immersive technology in older adults (Table 5). The results emphasize that, when compared to conventional repetitive treatment, immersive technology allows for greater interest, enjoyment, and motivation [16,39,42,57,64]. In addition, different authors [30,39,64] reported that participants, namely older people with mild to moderate dementia, people with Parkinson’s disease, or healthy older people had a pleasant experience with the VR. This can be explained by the feelings of relaxation and adventure that were present, as well as the reduction in anxiety, stress, and pain that was observed after exposure. This hypothesis is supported by recent studies demonstrating a stress and anxiety reduction among adults immersed into the VR environment [85,86]. Furthermore, a high level of interest and excitement about the VR technology before trying may have also contributed to these positive feelings reported after immersion. For instance, Appel et al. [30] and Brown [33] found that participants in their study wanted to use the immersive technology again in the future and would recommend it to a friend (Table 5). In most cases, the participants said that the headset they used (e.g., HTC Vive) was comfortable [64]. However, further studies are needed to confirm the acceptability of different types of immersive technology devices.

During the immersive experiences, some studies have focused on the environments that older people preferred to visit. Appel et al. [30] and Brown [33] showed that older people were interested in dynamic, social, and familiar real-world scenes (e.g., real places in the world, past or present). The authors suggested that the geriatric population would like to share these experiences with loved ones such as their grandchildren for narrative purposes or in order to explore places they no longer have the physical or psychological capacity to visit [33]. In addition to exploration and tourism, including mental relaxation, it should be noted that older people would also be open to other experiences with VR [64]. However, the environment in which a user is navigating significantly influences his or her desire to use VR [32].

Contrastingly, most commercial applications could be too complex and difficult to be used by older adults [39], especially for those with less experience with new technologies [33]. This may have decreased the acceptability of such devices in this population [64] and therefore may make further experiences less enjoyable. Moreover, there could be an increased feeling of isolation and loneliness for some people with physical or cognitive limitations [33]. Those feelings could subsequently promote depressive or anxious feelings and thus produce the opposite of the desired effect. Nevertheless, as suggested by Brown in their study [33], these concerns can be addressed with users prior to experimentation in an immersive environment.

### 4.2. Feasibility

The most commonly reported measure for determining the feasibility of a VR technology was the use of the Simulator Sickness Questionnaire (SSQ) [83]. The SSQ was developed to measure sickness that can occur when using VR technology (Table 6). It consists of side effects similar to those of motion-induced sickness [87]. These side effects may be caused by the visual conflict created by the immersive headset [32]. For example, after VR exposure, it has been reported that a side effect such as postural instability could significantly affect the Mini-BESTest score [46]. Moreover, owing to their medication or non-motor symptoms related to their condition [46], people with Parkinson’s disease may have a higher score on the SSQ questionnaire even before immersion [42]. Thereby, the use of immersive technology can generate increasing variation in the participants’ scores. However, some studies [46,55] showed that these changes were generally mild (with transient symptoms such as nausea, eye discomfort, disorientation, etc.) or not significant; although, in young and healthy adults, a longer duration of exposure seems to lead to more intense symptoms [88]. Further research would therefore be needed to generalize these observations to the geriatric population.

People with physical limitations may have to make more effort to succeed in virtual task completion, potentially limiting the treatment adherence and inducing some stress [40]. In addition, although dynamic activities such as walking seem to reduce the symptoms among healthy young adults [88], it is worth noting that for an older person performing walking movements during VR immersion, there is a higher risk of feeling stress [33,42,55,56]. Consequently, a familiarization period with the VR or AR equipment might be recommended prior to the interventions. This would ensure the comfort and feasibility of the experience and limit the unpleasant effects [33]. Moreover, the decrease in vision loss that occurs with ageing might be another barrier to the use of VR. Nevertheless, studies have provided recommendations for its use in people with vision loss [89,90]. First, VR applications should offer their users the possibility to modify the virtual visual field and light intensity according to their vision possibilities. Second, visual cues can be provided during the game to direct users’ attention towards important information that would be displayed in their affected field of view. Lastly, the use of prism in AR should be considered to optically shift objects from outside the vision field.

The types of headsets used (Table 1) are essential during an immersive experience since they can have a great impact on the occurrence of side effects. Indeed, modern headsets such as the Oculus Rift or the HTC Vive can decrease the occurrence and severity of the side effects due to a better refresh rate, larger field-of-view, and better head tracking compared to older or lower quality immersive headsets [16,46]. This may result in less intense and transient symptoms. However, the use of controllers is challenging for older people, especially if they are not familiar with the new technologies [33]. Additional difficulties that can negatively affect the use of immersive technology comprise the controller’s calibration and connection with the headset [33]. To overcome these difficulties, several systems have now developed hand-tracking technology, which allows for the use of a VR headset without using controllers. Indeed, hand-tracking enables one to generate a virtual model of hands and fingers into the VR environment by recording and identifying the movements of these body parts using infrared cameras. These methods have already been used and validated among patients with stroke and healthy older adults [91].

### 4.3. Effectiveness

The most important results of this systematic review also concern the effectiveness of the VR technology among the community-dwelling older adults (Table 7). The results on the effectiveness can be summarized in three main aspects.

First, as shown in Table 7 and Figure 2, VR can be used to improve balance in older people and reduce the risk of falls. Indeed, this technology could achieve results similar to conventional exercises, but in half the time, with an intensity of 2 sessions of 30 min per week for 6 weeks in healthy subjects [57]. A significant improvement was also observed with the Mini-BESTest scores in people with Parkinson’s disease [46], as well as the Activities-specific Balance Confidence (ABC) Scale and the TUG among participants with stroke [44]. VR can also promote a more personalized approach for the user, allowing for greater specificity in the treatment of balance deficits, thus improving gains and adherence [57]. In fact, the VR method proposed in [51,56] has been shown to significantly decrease anterior trunk rotations, keeping the center of mass within the base of stability and thus reducing the incidence of falls [92]. By using the Falls Efficacy Scale (FES-I) in a study involving healthy subjects, Phu et al. [57] showed that the use of VR led to a small but significant decrease in the fear of falling.

Second, VR can be used to correct the gait pattern. Indeed, thanks to the screen embedded in the headset, the participant’s virtual foot appears to take a larger step in contrast to reality, exaggerating the decrease in the step length on the more affected side and thus forcing the user to take more symmetric steps on both sides [42]. Thus, the stance and swing times appeared to be more symmetrical after exposure to VR. This leads to the regularization of the cadence of the more affected side and a more symmetrical overall gait pattern [42]. However, larger randomized studies over a longer period are needed to confirm this effectiveness.

Third, Benham et al. [32] have shown a significant (*p* < 0.05) decrease in pain among participants after one session of VR. This decrease in the pain can be attributed to the distraction provided by the immersion. In fact, several studies have suggested that the immersive aspect of VR might be responsible for the reduced subjective experience of pain as the interaction with the real-world cues are being limited by the use of HMD [93]. This effect might be enhanced by the engaging, pleasant, and multisensorial feature of the immersive VR environment [93].

The effect of VR was also shown for other outcomes. VR resulted in fine motor skills improvements in a group of participants with Parkinson’s and a slight improvement in the Unified Parkinson’s Disease Rating Scale (UPDRS) [39]. An improvement in the grip strength was also observed in healthy subjects with the use of BRU [41]. Micarelli et al. [53] concluded that the addition of VR resulted in a significant improvement of the vestibulo-ocular reflex by increasing the frequency of visuo-vestibular conflicts. However, this study was conducted with a small sample of older people with mild cognitive impairments.

### 4.4. Perspectives

The results reported in this work provide several perspectives for the use of immersive technologies among older adults. While these technologies (VR and AR) are not yet implemented in our daily life, their increasing popularity, the decrease in their price, and their potential in terms of realism, interaction, and communication might reverse the situation. For instance, with the development of the metaverse, a parallel immersive virtual world intended to supplant the internet, it could be that rehabilitation is delivered remotely more often [94,95]. Such environments might also be of interest to improve older people’s social participation as it allows for realistic multi-user interactions. Given such perspectives, we believe that, in the future, VR will be used as a mean to deliver effective remote rehabilitation to complement therapy and increase treatment adherence and intensity.

Moreover, VR devices (and metaverse development) also hold potential to deliver rehabilitation and promote activity among people who have no/few access to healthcare services, such as in low-income countries. Several studies have demonstrated the feasibility of implementing such interventions in developing countries [96,97].

### 4.5. Limitations

Despite the positive effects of VR reported in different studies and summarized here, the generalization of these results ais limited by the small number of articles available for each population and for each type of outcome, as discussed above. The overall low methodological quality of the articles included in this review potentially reduces the strength of the reported conclusions. Despite these limitations, this first systematic review shows encouraging results for further research and decisions in clinical settings.

## 5. Conclusions

Virtual reality is well accepted by older people and provides an enjoyable experience. The results suggest that the use among this population is feasible since few symptoms were reported and the increased SSQ scores were not significant in most cases. Currently, despite the several advantages described above, it is impossible to conclude on the effectiveness of VR in relation to different pathologies and deficiencies since few studies with good methodological quality and sufficiently large sample sizes are available. However, the beneficial effects have been observed regarding balance, risk of falling, and gait pattern in studies with acceptable methodological quality.

## Figures and Tables

**Table 1:** Characteristics of the included studies.

Author(s), Year	Country	Participants’ Group and Average Age (Years)			Average Time Since Diagnostic	Severity of Illness	*n*	♂/♀	Study Design	Exposure Duration	Supervision	Headset Used	Acceptability	Feasibility	Effectiveness
Immersive virtual reality															
Appel et al., 2020 [30]	Canada	Mild to moderate dementia (80.5 + 10.5) Subgroup Baycrest 80.5 ± 10.5 79.5 ± 9.1			N/A	MOCA, CPS, and MMSE: Normal = 28 Mild = 17 Moderate = 12 Severe = 3 Unknown = 6	66	26/40	Experimental	1 × 3 to 20 min	Yes	Samsung Gear VR	X	X	
		Group 1			N/A	MOCA: Normal = 7 Mild = 8 Moderate = 2 Severe = 0 Unknown = 1	18	9/9							
		Group 2	Subgroup Kensington	80.7 ± 11.7	N/A	CPS: Normal = 16 Mild = 8 Moderate = 9 Severe = 0	33	12/21							
		Group 3	Subgroup Runnymede	82.7 ± 10.1	N/A	MMSE: Normal = 5 Mild = 1 Moderate = 1 Severe = 3	10	4/6							
		Group 4	Subgroup Bitove	78.7 ± 8.8	N/A	MOCA, CPS, and MMSE: Unknown = 5	5	1/4							
Barsasella et al., 2021 [31]	Taiwan	Group 1	VR sessions	>60	N/A	N/A	29	4/25	Randomized controlled trial	12 × 15 min	Yes	HTC Vive		X	X
		Group 2	No sessions	>60	N/A	N/A	31	10/21							
Benham et al., 2019 [32]	United States	Total	Pain	70.2 ± 3.6	N/A	Pain that interferes with daily activities	12	4/8	Experimental	12 × 15 to 45 min	Yes	HTC Vive	X		X
Brown, 2019 [33]	United States	Total	Healthy	63 to 89	N/A	N/A	10	2/8	Qualitative study	N/A	N/A	Samsung Gear VR	X	X	
Burin et al., 2021 [34]	Japan	Group 1	First person perspective	70.5 ± 6.5	N/A	N/A	21	10/11	Randomized controlled trial	12 × 20 min	Yes	Oculus Rift		X	X
		Group 2	Third person perspective	72.9 ± 4.6	N/A	N/A	21	4/17							
Campo-Prieto et al., 2021 [35]	Spain	Total	Healthy	70.8 ± 5.7	N/A	N/A	4	4/0	Experimental	2 × 6 min	Yes	HTC Vive		X	
Campo-Prieto et al., 2022 (a) [36]	Spain	Total	Healthy	71.5 ± 11.8	6 years	Hoehn and Yahr scale: Level 2	32	25/7	Experimental	Not mentioned	Yes	Oculus Quest	X	X	
Campo-Prieto et al., 2022 (b) [37]	Spain	Group 1	Usual care + VR training	91.7 ± 1.6	N/A	N/A	6	0/6	Randomized controlled trial	(10 × 45) + (30 × 6 min)	Yes	HTC Vive		X	X
		Group 2	Usual care	90.8 ± 2.6	N/A	N/A	6	0/6							
Campo-Prieto et al., 2022 (c) [38]	Spain	Group 1	Usual care + VR intervention	85.1 ± 8.5	N/A	N/A	13	2/11	Randomized controlled trial	30 × 6	Yes	HTC Vive		X	X
		Group 2	Usual care	84.8 ± 8.1	N/A	N/A	11	1/10							
Cikajlo and Peterlin Potisk, 2019 [39]	Slovenia	Total	Parkinson’s	N/A	7.1 years	Hoehn and Yahr scale: Levels 2–3	20	9/11	Randomized parallel study	10 × 30 min	Yes	Oculus Rift CV1	X	X	X
		Group 1	Parkinson’s VR	67.6 ± 7.6	N/A	N/A	10	5/5							
		Group 2	Parkinson’s LCD	71.3 ± 8.4	N/A	N/A	10	4/6							
Crosbie et al., 2006 [40]	Ireland	Group 1	Stroke	62	10 years	N/A	5	N/A	Experimental with control group	N/A	N/A	UUJ VRR System		X	
		Group 2	Healthy adults (Control)	42	N/A	N/A	10	N/A							
De Keersmaecker et al., 2020 [16]	Belgium	Total	Healthy	N/A	N/A	N/A	28	13/15	Randomized controlled trial	21 min (3 walking conditions × 7 min)	Yes	Oculus Rift	X	X	
		Group 1	Walk in the park	61 ± 6	N/A	N/A	14	6/8							
		Group 2	Walk in a corridor	62 ± 5	N/A	N/A	14	7/7							
Hoeg et al., 2021 [41]	Denmark	Total	Healthy	60 ± 11	N/A	N/A	11	7/4	Experimental	10 to 15 min	Yes	Oculus Rift	X	X	
Janeh et al., 2019 [42]	Germany	Total	Parkinson	67.6 ± 7	9.5 years ± 4.9	Hoehn and Yahr scale: 2–3	15	15/0	Experimental	5–6 min	Yes	HTC Vive	X	X	X
Jang et al., 2020 [43]	South Korea	Group 1	VR-based cognitive training	72.6 ± 5.4	Not mentioned	MMSE: 26 ± 1.8	34	6/28	Randomized controlled trial	24 × 100 min	Yes	Oculus Quest			X
		Group 2	Educational program	72.7 ± 5.6	Not mentioned	MMSE: 26.3 ± 3.3	34	10/24							
Jung et al., 2012 [44]	South Korea	Group 1	Stroke (Experimental group	60.5 ± 8.6	12.6 ± 3.3 (months)	Able to walk more than 30 min	11	7/4	Randomized controlled trial	15 × 30 min	N/A	HMD (Brand N/A)			X
		Group 2	Stroke (Control group)	63.6 ± 5.1	15.4 ± 4.7 (months)	Able to walk more than 30 min	10	6/4							
Kanyilmaz et al., 2021 [45]	Turkey	Group 1	Vestibular rehabilitation supported with VR	70 ± 6	>3 months	VVS: 9 ± 11	13	6/7	Randomized controlled trial	15 × 30 min	Yes	Samsung Gear VR			X
		Group 2	Conventional vestibular rehabilitation	70 ± 5	>3 months	VVS: 15 ± 18	13	4/9							
Kim et al., 2017 [46]	United States	Group 1	Healthy	66 ± 3	N/A	MOCA: 27 ± 2	11	3/8	Experimental	20 min	Yes	Oculus Rift DK2		X	X
		Group 2	Parkinson	65 ± 7	7 (1–32)	MOCA: 26 ± 3	11	3/8							
Kiper et al., 2022 [47]	Poland	Group 1	Immersive VR therapeutic garden	65.5 ± 6.7	3.9 ± 1.5	MMSE: 26.4 ± 2.3	30	13/17	Randomized controlled trial	(10 × 60) + 10 × 20 min	Yes	HTC Vive			X
		Group 2	Schultz’s autogenic training	65.6 ± 5	4 ± 1.5	MMSE: 27.2 ± 1.5	30	17/13							
Kruse et al., 2021 [48]	Germany	Total	Healthy	81.2 ± 5	N/A	N/A	25	3/22	Experimental	7–10 min	Yes	Valve Index	X	X	
Li et al., 2020 [49]	Japan	Group 1	VR intervention	73.8 ± 7.4	N/A	N/A	10	3/7	Randomized controlled trial	12 × 45 min	Not mentioned	HTC Vive	X		X
		Group 2	No intervention	72.4 ± 7.8	N/A	N/A	10	4/6							
Liepa et al., 2022 [50]	Latvia	Group 1	Immersive VR-based intervention	72.4 5.9	N/A	N/A	14	4/10	Randomized controlled trial	18 × 20 min	Yes	HTC Vive	X		X
		Group 2	Non-immersive VR-based intervention	73.1 6.3	N/A	N/A	15	2/13							
		Group 3	Usual activities	71.7 6	N/A	N/A	15	3/12							
Liu et al., 2015 [51]	United States	Total	Healthy	N/A	N/A	N/A	24	N/A	Experimental	N/A	Yes	Glasstron LDI-100B			X
		Group 1	Virtual reality	70.54 ± 6.63	N/A	N/A	12	N/A							
		Group 2	Control	74.18 ± 5.82	N/A	N/A	12	N/A							
Matamala-Gomez et al., 2022 [52]	Spain	Group 1	Immersive VR	60.1 ± 12.8	Not mentioned	85% with UE FMA > 57	20	0/20	Randomized controlled trial	4 to 6 × 3 × 20 min	Yes	Oculus Rift	X		X
		Group 2	Conventional digital mobilization	61.1 ± 16.2	Not mentioned	25% with UE FMA > 57	20	3/17							
		Group 3	Non-immersive VR	64.6 ± 13.5	Not mentioned	0% with UE FMA > 57	14	5/9							
Micarelli et al., 2019 [53]	Italy	Total	Unilateral vestibular hypofunction	75.7 ± 4.8	N/A	N/A	23	11/12	Randomized controlled trial	N/A	N/A	Revelation 3D VR Headset + Lumia 930		X	X
		Group 1	VR headset + vestibular rehabilitation	76.9 ± 4.7	17.2 ± 6	N/A	11	5/6							
		Group 2	Vestibular rehabilitation	74.3 ± 4.7	16.5 ± 5.7	N/A	12	6/6							
Muhla et al., 2020 [54]	France	Total	Healthy	73.7 ± 9	N/A	N/A	21	N/A	Experimental	N/A	Yes	HTC Vive		X	
		Group 1	Healthy with TUG VR	N/A	N/A	N/A	N/A	N/A							
		Group 2	Healthy with TUG	N/A	N/A	N/A	N/A	N/A							
Parijat and Lockhart, 2011 [55]	United States	Total	Healthy	74.18 ± 5.82	N/A	N/A	16	8/8	Experimental	5 to 25 min	Yes	Glasstron LDI-100B		X	X
Parijat et al., 2015 [56]	United States	Total	Healthy	>65	N/A	N/A	24	12/12	Randomized controlled trial	N/A	Yes	Glasstron LDI-100B			X
		Group 1	Virtual reality training	70.5 ± 6.6	N/A	N/A	12	N/A							
		Group 2	Control	74.2 ± 5.8	N/A	N/A	12	N/A							
Phu et al., 2019 [57]	Australia	Total	Healthy	78 (73–84)	N/A	N/A	195	65/130	Experimental controlled trial	12 × 15 min	Yes	BRU	X		X
		Group 1	BRU	79 (74–84)	N/A	N/A	63	19/44							
		Group 2	Balance exercises without VR	76 (71–82)	N/A	N/A	82	31/51							
		Group 3	Control	79 (72–82)	N/A	N/A	50	15/35							
Rebelo et al., 2022 [58]	Brazil	Group 1	VR-based balance training	69.3 ± 5.7	Not mentioned	DGI: 18.2 ± 3.9	20	4/16	Randomized controlled trial	16 × 50 min	Yes	Oculus Rift			X
		Group 2	Conventional balance training	71.4 ± 5.9	Not mentioned	DGI: 15.3 ± 3.7	17	2/15							
Rutkoswki et al., 2021 [59]	Poland	Group 1	Pulmonary rehabilitation + VR-based relaxation	64.4 ± 5.7	N/A	N/A	25	4/21	Randomized controlled trial	10 × 15–30 min	Yes	HTC Vive			X
		Group 2	Pulmonary rehabilitation + Schultz’s autogenic training	67.6 ± 9.4	N/A	N/A	25	5/20							
Sakhare et al., 2021 [60]	United States	Total	Healthy	64.7 ± 8.8	N/A	N/A	20	12/8	Experimental	35 × 25–50 min	Yes	HTC Vive			X
Saldana et al., 2017 [61]	United States	Group 1	At risk of falling	78.4 ± 9.37	N/A	N/A	5	2/3	Experimental	2 visits, time N/A	N/A, but security system present	Oculus Rift DK2		X	
		Group 2	Low risk of falling (Control)	81.4 ± 6.25	N/A	N/A	8	1/7							
Stamm et al., 2022 (a) [62]	Germany	Total	Older hypertensive	75.4 ± 3.6	Not mentioned	Not mentioned	22	9/13	Experimental	2 × 25 min	Yes	HTC Vive	X	X	
Stamm et al., 2022 (b) [63]	Germany	Group 1	VR-based rehabilitation	75 ± 5.8	15.8 ± 18.7 years	NRS: 3.4 ± 1.9	11	3/8	Randomized controlled trial	12 × 30 min	Yes	HTC Vive		X	X
		Group 2	Group exercise	75.5 ± 4.4	26.4 ± 16.6 years	NRS: 2.9 ± 1.6	11	5/6							
Syed-Abdul et al., 2019 [64]	Taiwan	Total	Healthy	>60	N/A	N/A	30	6/24	Qualitative study (Technology Acceptance Model)	12 × 15 min	N/A	HTC Vive	X	X	
Szczepanska-Gieracha et al., 2021 [65]	Poland	Group 1	Fitness, Psychoeducation + VR	70.2 ± 4.9	Not mentioned	GDS: 12.3 ± 4.5	11	0/11	Randomized controlled trial	8 × 60 min	Yes	HTC Vive			X
		Group 2	Fitness and Psychoeducation	71.2 ± 4.4	Not mentioned	GDS: 12.3 ± 4.5	12	0/12							
Valipoor et al., 2022 [66]	United States	Group 1	Healthy	72.6 ± 6.4	N/A	N/A	24	11/13	Experimental	Not mentioned	Yes	HTC Vive	X	X	
		Group 2	Parkinson	72.7 ± 6	<5 years	Hoehn and Yahr scale I-III	15	9/6							
Vieira et al., 2020 [67]	United States	Total	Healthy	68 ± 5	N/A	N/A	10	Not mentioned	Experimental	One session	Yes	HTC Vive and Microsoft HoloLens (AR)	X		
Yalfani et al., 2022 [68]	Iran	Group 1	VR-based intervention	68 ± 2.9	Not mentioned	LBP VAS: 6.7 ± 2.4	13	0/13	Randomized controlled trial	24 × 30 min	Yes	HTC Vive			X
		Group 2	No treatment	67.1 ± 2.9	Not mentioned	LBP VAS: 6.8 ± 2	12	0/12							
Yang et al., 2022 [69]	South Korea	Group 1	VR-based intervention	72.5 ± 5	Not mentioned	MMSE: 27.2 ± 1.9	33	13/20	Randomized controlled trial	24 × 100 min	Yes	Oculus Quest			X
		Group 2	Exercise training	68 ± 3.6	Not mentioned	MMSE: 26.9 ± 1.7	33	3/30							
		Group 3	Education seminars	67.1 ± 2.9	Not mentioned	MMSE: 26.5 ± 2.8	33	6/27							
Yoon et al., 2020 [70]	South Korea	Group 1	Passive motion therapy + VR	72.2 ± 3.7	Day 0	N/A	18	0/18	Randomized controlled trial	(10 × 30) + 10 × 20 min	Yes	VR GLASS			X
		Group 2	Passive motion therapy	71.8 ± 4.9	Day 0	N/A	18	0/18							
Zak et al., 2022 [71]	Poland	Group 1	VR-based rehabilitation room + conventional therapy	79.1 ± 3.6	Not mentioned	IADL: 20.3 ± 2.3	15	24/36	Randomized controlled trial	9 × 60 min	Yes	Oculus Rift			X
		Group 2	Dual task training + VR	78.1 ± 3.7	Not mentioned	IADL: 19.3 ± 1.4	15								
		Group 3	VR alone (maze game)	76.7 ± 1.5	Not mentioned	IADL: 19.7 ± 1.9	15								
		Group 4	Conventional therapy	76.7 ± 1.6	Not mentioned	IADL: 19.3 ± 2	15								
Augmented reality															
Bank et al., 2018 [72]	Netherlands	Group 1	Healthy	61.6 ± 6.8	N/A	N/A	10	6/4	Experimental	N/A	Yes	AIRO II		X	X
		Group 2	Parkinson	60.8 ± 7.5	11.9 (7.4–15.7)	Hoehn and Yahr: 2 (1–3)	10	6/4							
		Group 3	Stroke	60.5 ± 7.0	3.5 (1.9–9.1)	Fugl-Meyer: 59.5 (55.8–64)	10	6/4							
Cerdan de las Heras et al., 2020 [73]	Finland	Total	Healthy	63.8	N/A	N/A	13	11/2	Qualitative	Not mentioned	Yes	Laster WAVƎ	X		
Chen et al., 2020 [74]	Taiwan	Group 1	AR-assisted Tai Chi	72.2 ± 2.8	N/A	N/A	14	2/12	Randomized controlled trial	24 × 30 min	Yes	Microsoft Kinect			X
		Group 2	Traditional Tai Chi	75.1 ± 5.5	N/A	N/A	14	1/13							
Ferreira et al., 2022 [75]	Portugal	Total	Healthy	72 ± 5.2	N/A	N/A	27	18/9	Experimental	(2 × 30) + (1 × 30 min)	Yes	Portable Exergame Platform for Elderly (PEPE)			X
Fischer et al., 2007 [76]	United States	Total	Chronic Stroke	60 ± 14	7 ± 9	Chedoke-McMaster Stroke Assessment (Hand Subscale): Stage 2–3 Fugl-Meyer for upper member: 24 ± 11	15	9/6	Randomized controlled trial	18 × 1 h (time in virtual reality vs. real reality N/A)	Yes	Glasstron PLM-5700			X
		Group 1	Pneumatic orthosis	71.60 ± 13.86 †	4.45 ± 2.90 †	18.60 ± 9.07 †	5	4/1							
		Group 2	Wired orthosis	53.00 ± 12.21 †	6.40 ± 4.39 †	28.00 ± 23.22 †	5	2/3							
		Group 3	Control Group	55.60 ± 9.94 †	11.20 ± 15.22 †	25.20 ± 5.54 †	5	3/2							
Jeon et al., 2020 [77]	South Korea	Group 1	AR-based exercises	72.8 ± 3.8	N/A	N/A	13	0/13	Randomized controlled trial	60 × 30 min	Yes	UNICARE HEALTH			X
		Group 2	No intervention	72.7 ± 3.6	N/A	N/A	14	0/14							
Koroleva et al., 2020 [78]	Russia	Group 1	Traditional rehabilitation + AR	62 [57–67]	Subacute	FMA LE: 24 [21–27]	21	13/8	Controlled study	Not specifically mentioned	Yes	NEURO RAR			X
		Group 2	Only AR-based rehabilitation	65.5 [60–68]	Subacute	FMA LE: 26 [21–28]	14	7/7							
		Group 3	No intervention	66 [60.5–68]	Subacute	FMA LE: 24 [20–29]	15	8/7							
		Group 4	Healthy	63 [56–65]	N/A	N/A	50	29/21							
Koroleva et al., 2021 [79]	Russia	Group 1	Conventional rehabilitation + AR	62 [57–67]	Subacute	NIHSS: 5 [3–6]	21	13/8	Controlled study	Daily session of 60 min	Yes	NEURO RAR			X
		Group 2	Very early rehabilitation + AR	65 [60–68]	Subacute	NIHSS: 5 [4–7]	14	7/7							
		Group 3	Very early rehabilitation	66 [60.5–68]	Subacute	NIHSS: 6 [3–8]	15	8/7							
Munoz et al., 2021 [80]	Spain	Total	Healthy	65–80	N/A	N/A	57	29/26	Experimental	6 sessions	Yes	Microsoft Kinect	X		X
Vieira et al., 2020 [67]	United States	Total	Healthy	68 ± 5	N/A	N/A	10	Not mentioned	Experimental	1 session	Yes	Microsoft HoloLens and HTC Vive (VR)	X		
Yoo et al., 2013 [81]	South Korea	Total	Healthy	N/A	N/A	N/A	21	N/A	Randomized controlled trial	N/A	N/A	i-visor FX601			X
		Group 1	Virtual reality training	72.9 ± 3.41	N/A	N/A	10	N/A							
		Group 2	Training without VR	75.64 ± 5.57	N/A	N/A	11	N/A							
CAVE															
Pedroli et al., 2018 [82]	Italy	Total	Healthy	70.00 ± 11.70	N/A	N/A	5	2:3	Qualitative	15 min	Yes	CAVE (Ø HMD)	X	X	

MOCA: Montreal Cognitive Assessment; VR = virtual reality; N/A = not available; CAVE = cave automatic virtual environment. †: Computed by the authors of this systematic review and not by the authors of the referenced article.

**Table 2:** PEDro scale rating for experimental articles.

Author(s)	Year	Score (/8)	a	b	c	d	e	f	g	h	i
Immersive virtual reality											
Barsasella et al. [31]	2021	6	+	+	+	+	−	+	−	+	+
Burin et al. [34]	2021	6	+	+	+	+	−	+	−	+	+
Campo-Prieto et al. (b) [37]	2022	6	+	+	−	+	−	+	+	+	+
Campo-Prieto et al. (c) [38]	2022	6	+	+	+	+	−	+	−	+	+
Cikajlo et al. [39]	2019	6	+	+	−	+	−	+	+	+	+
De Keersmaeker et al. [16]	2020	4	−	−	−	−	−	+	+	+	+
Jang et al. [43]	2020	7	+	+	+	+	−	+	+	+	+
Jung et al. [44]	2012	6	+	+	−	−	+	+	+	+	+
Kanyilmaz et al. [45]	2022	4	+	+	−	+	−	−	−	+	+
Kiper et al. [47]	2022	7	+	+	+	+	+	−	+	+	+
Li et al. [49]	2020	5	+	+	−	−	−	+	+	+	+
Liepa et al. [50]	2022	5	+	+	+	+	−	−	−	+	+
Liu et al. [51]	2015	4	−	−	−	−	−	+	+	+	+
Matamala-Gomez et al. [52]	2022	6	+	+	+	+	−	−	+	+	+
Micarelli et al. [53]	2019	6	+	+	−	+	−	+	+	+	+
Parijat et al. [55]	2015	5	−	−	−	+	−	+	+	+	+
Phu et al. [57]	2019	4	+	−	−	−	−	+	+	+	+
Rebelo et al. [58]	2021	7	+	+	+	+	+	+	−	+	+
Rutkowski et al. [59]	2021	8	+	+	+	+	+	+	+	+	+
Stamm et al. (b) [63]	2022	8	+	+	+	+	−	+	+	+	+
Szczepanska-Gieracha et al. [65]	2021	5	+	+	−	+	−	+	−	+	+
Yalfani et al. [68]	2022	4	+	+	−	+	−	−	−	+	+
Yang et al. [69]	2022	6	+	+	−	+	−	+	+	+	+
Yoon et al. [70]	2020	6	+	+	+	+	+	−	−	+	+
Zak et al. [71]	2022	5	−	+	−	+	−	+	−	+	+
Augmented reality											
Chen et al. [74]	2020	6	+	+	−	+	+	+	−	+	+
Fischer et al. [76]	2007	5	−	+	−	−	−	+	+	+	+
Jeon et al. [77]	2020	6	+	+	+	+	−	+	−	+	+
Koroleva et al. [78]	2020	5	+	−	−	+	−	+	+	+	+
Koroleva et al. [79]	2021	5	+	−	−	+	−	+	+	+	+
Yoo et al. [81]	2013	4	+	−	−	−	−	+	+	+	+

The listed criteria below is Present (+) or Absent (−): a = the eligibility criteria have been specified; b = participants were randomly assigned to the groups; c = the assignment of participants to a group was concealed; d = at the beginning of the study, the groups were similar; e = evaluators who measured at least one key outcome did not know which group the participants were assigned to; f = measures of at least one key outcome were obtained in more than 85% of participants initially assigned to the groups; g = all participants for whom outcome measures were available received the assigned intervention; h = results of inter-group statistical comparisons are provided for at least one key outcome; i = the study provides both an effect size measure and a measure of dispersion for at least one key outcome.

**Table 3:** NIH Quality Assessment tool.

Author(s)	Year	a	b	c	d	e	f	g	h	i	j	k	l	m	n
Immersive virtual reality															
Appel et al. [30]	2020	+	+	~	+	−	~	−	+	+	+	+	−	+	−
Benham et al. [32]	2019	+	+	−	+	−	~	+	−	+	+	+	−	+	−
Campo-Prieto et al. [35]	2021	+	+	−	−	−	~	−	−	+	+	+	−	+	−
Campo-Prieto et al. (a) [36]	2022	+	+	−	+	−	~	~	−	+	+	+	−	+	−
Crosbie et al. [40]	2006	~	+	−	+	−	~	~	−	+	~	~	−	+	−
Hoeg et al. [41]	2021	+	~	−	+	−	~	−	−	+	−	+	−	+	−
Janeh et al. [42]	2019	+	+	−	+	−	~	−	−	+	−	+	+	+	+
Kim et al. [46]	2017	+	−	−	−	−	~	−	−	+	−	+	+	+	+
Kruse et al. [48]	2021	+	~	−	−	−	~	−	−	+	−	+	−	+	−
Muhla et al. [54]	2015	+	−	−	−	−	~	~	~	+	~	+	+	+	−
Parijat et al. [55]	2011	+	+	−	~	−	~	−	+	+	−	+	−	+	−
Sakhare et al. [60]	2021	+	+	−	+	−	~	+	−	+	+	+	−	−	~
Saldana et al. [61]	2017	~	+	−	+	−	~	−	+	+	+	+	−	−	−
Stamm et al. (a) [62]	2022	+	+	−	+	−	~	+	−	+	+	+	−	−	+
Valipoor et al. [66]	2022	+	+	−	−	−	~	−	+	+	−	+	−	+	+
Vieira et al. [67]	2020	+	+	−	~	−	~	−	−	+	−	+	−	+	−
Augmented reality															
Bank et al. [72]	2018	~	−	−	−	−	~	~	+	+	~	+	−	−	−
Ferreira et al. [75]	2022	+	+	−	−	−	~	+	−	+	+	+	−	−	+
Munoz et al. [80]	2021	+	−	−	−	−	~	+	−	+	+	+	−	−	+
Vieira et al. [67]	2020	+	+	−	~	−	~	−	−	+	−	+	−	+	−

+ = Yes; − = No; ~ = Uncertain; a = Was the research question or objective in this paper clearly stated?; b = Was the study population clearly specified and defined?; c = Was the participation rate of eligible persons at least 50%?; d = Were all the subjects selected or recruited from the same or similar populations (including the same time period)? Were inclusion and exclusion criteria for being in the study prespecified and applied uniformly to all participants?; e = Was a sample size justification, power description, or variance and effect estimates provided?; f = For the analyses in this paper, were the exposure(s) of interest measured prior to the outcome(s) being measured?; g = Was the timeframe sufficient so that one could reasonably expect to see an association between exposure and outcome if it existed?; h = For exposures that can vary in amount or level, did the study examine different levels of the exposure as related to the outcome (e.g., categories of exposure or exposure measured as continuous variable)?; i = Were the exposure measures (independent variables) clearly defined, valid, reliable, and implemented consistently across all study participants?; j = Was the exposure(s) assessed more than once over time?; k = Were the outcome measures (dependent variables) clearly defined, valid, reliable, and implemented consistently across all study participants?; l = Were the outcome assessors blinded to the exposure status of participants?; m = Was loss to follow-up after baseline 20% or less?; n = Were key potential confounding variables measured and adjusted statistically for their impact on the relationship between exposure(s) and outcome(s)?

**Table 4:** CEBM scale rating for qualitative studies.

Author(s)	Year	a	b	c	d	e	f	g	h
Immersive virtual reality									
Brown et al. [33]	2019	+	+	+	+	−	+	+	−
Syed-Abdul et al. [64]	2019	+	+	+	+	+	+	+	−
Augmented reality									
Cerdan des las Heras et al. [73]	2020	+	~	+	~	+	+	+	−
CAVE									
Pedroli et al. [82]	2018	+	~	+	+	−	+	+	−

CAVE = cave automatic virtual environment; CEBM = centre for evidence-based medicine; + = Yes; ~ = Uncertain; − = No; a = Was the qualitative approach appropriate?; b = Was the sampling strategy appropriate?; c = What is the method of data collection?; d = How was the data analyzed?; e = Was the researcher’s position described?; f = Do the results make sense and are they credible?; g = Are the conclusions justified by the results?; h = Are the findings transferable to other clinical settings?

**Table 5:** Results and author’s conclusions on the acceptability of immersive technologies with a geriatric population.

Author(s), Year	Data Collection Method		Results		*p*-Value	Author(s)’ Conclusions
Immersive virtual reality						
Appel et al., 2020 [30]	Questionnaire	Pleasure during the activity	13 = None 7 = A little 22 = Moderate 18 = A lot		N/A	Generally considered to be pleasant. Would like to do it again. Would recommend it to someone else.
		Discussion that shows interest	13 = None 7 = A little 11 = Moderate 22 = A lot		N/A	
		Facial expression during virtual reality that indicates awareness of the experience	11 = None 17 = A little 20 = Moderate 16 = A lot		N/A	
Benham et al., 2019 [32]	Open written questionnaire		Positive experience: 100% Positive effect on pain levels: 100% Would continue to use virtual reality if given the chance: 91.7% Would recommend the device to other users in the residence: 100% Experienced negative symptoms while using VR (e.g., nausea, headaches, eye strain): 41.7%		N/A	Participants were very enthusiastic. VR is enjoyable with the elderly. Immersive VR can cause side effects. It is therefore recommended to have proper supervision and monitoring when used with the elderly.
Brown, 2019 [33]	Interviews and focus groups		Older people with experience with digital platforms needed less guidance. Experience would have been more enjoyable with music. Enjoyed seeing places in the present, but would also enjoy seeing places in the past or visiting places that they would not have the capacity to do so today. Should be able to share this experience with others and not just do it alone for storytelling and socialization. Could help those with cognitive or physical limitations. Could have 3D meetings with family members or friends. Could increase feelings of isolation, anxiety, and depression in some people who are physically limited.		N/A	Participants reported that they enjoyed the experience and would consider using VR again if given the opportunity. Good option for reliving certain experiences and for entertainment, exploration, education, and socialization. People with more experience with new technologies would find it easier to use virtual reality. Participants reported feeling safe at all times. VR can promote socialization if it allows for the incorporation of family and friends. May increase some feelings of isolation, anxiety, or depression in some people. Would benefit to discuss these concerns prior to use.
Campo-Prieto et al., 2021 [35]	System Usability Scale (SUS)		P1: 100/100 P2: 85–90/100 P3: 100/100 P4: 85–95/100		N/A	The answers on the usability, with the presence of no adverse events, underline the safety of the tool.
Campo-Prieto et al., 2022 (a) [36]	System Usability Scale (SUS)		75.2 ± 7.5		N/A	Patients showed high levels of user satisfaction.
Cikajlo and Peterlin Potisk, 2019 [39]	IMI	Q3 + Q7 (Interest/Agreeableness)	U3 [CI] = 0.5 [0.4–0.9]		*p* = 0.995 (between groups)	Better motivation in the immersive group, especially in time → finished the level faster and was more efficient but LCD group was more relaxed and made fewer mistakes.
		Q5 + Q8 (Effort/Importance)	U3 [CI] = 0.5 [0.0–1.0]		*p* = 0.418 (between groups)	
De Keersmaecker et al., 2020 [16]	Physical Activity Enjoyment Scale (PACES)		Parc = 92.14 ± 18.86 Corridor = 92.64 ± 15.73		N/A	The type of place in which the user travels has no effect on the rating. Experience appreciated in the two different environments
Hoeg et al., 2021 [41]	System Usability Scale (SUS)		85 ± 5		N/A	Participants globally agree that they would use the VR system frequently.
Janeh et al., 2019 [42]	System Usability Scale (SUS)		3.5 ± 0.8		N/A	Participants had a moderate sense of presence in VR. They rated their fear of running into physical obstacles while immersed in HMD as relatively low.
Kruse et al., 2021 [48]	Intrinsic Motivation Index (IMI)		Immersive exergame: 4.6 ± 0.6	Non-immersive exergame: 4.6 ± 0.7	*p* = 0.871 (between group)	Participants did enjoy the immersive exergame as much as the non-immersive.
Li et al., 2020 [49]	Intrinsic Motivation Index (IMI)		No change of motivation after 4 weeks of immersive VR-based training.		*p* > 0.05	Participants did enjoy the game and did not change their motivation after 4 weeks, suggesting its potential for long-term training.
Liepa et al., 2022 [50]	Open-ended questions		The VR game was perceived as motivating. The game was making a participant positive. The immersion was well received.		N/A	Participants were satisfied with the game although they provided some suggestions to improve the game.
Matamala-Gomez et al., 2022 [52]	Virtual reality experience questionnaire		Participants reported higher experience scores for immersive VR (when compared to non-immersive VR)		*p* < 0.001	Participants reported higher experience score for immersive VR
Phu et al., 2019 [57]	% adherence to the treatment		Exercises: 72% RV: 71%		N/A	The EX and RV groups had similar levels of adherence.
Stamm et al., 2022 (a) [62]	Technology Usage Inventory		No significant difference between the gamified VR app and the strength-endurance VR app.		*p* = 0.794	The acceptance did not differ between the guided instruction VR-SET and the gamified VR-ET exergame.
Syed-Abdul et al., 2019 [64]	Written questionnaire		Perceived usefulness = 3.80 ± 0.571–4.07 ± 0.583 User experience = 3.77 ± 0.626–4.07 ± 0.583 Intent to use: 3.63 ± 0.615–3.90 ± 0.607 Social norms: 3.43 ± 0.626–3.77 ± 0.626		N/A	Older people consider using a technology based on its ease of use and usefulness. In addition, enjoyment is an important element of the intention to use VR. Social norms also have a direct effect on the intention to use VR. Older people seemed to enjoy VR and found it useful in motivating them in their daily activities. VR was comfortable and provided a new and positive experience. Finally, older people had a positive perception of the usefulness of VR.
Valipoor et al., 2022 [66]	System Usability Scale (SUS)		Older adults 41.4 ± 6.6	Parkinson 43.3 ± 7.2	N/A	Participants were satisfied with the system and found the tool usable.
	User Satisfaction Scale (USEQ)		Older adults 76.1 ± 13.6	Parkinson 78.9 ± 5.5		
Augmented reality						
Cerdan de las Heras et al., 2020 [73]	Interviews and focus groups		AR was seen as a natural experience that can be performed indoor and outdoor. Wearing AR glasses should be comfortable. A 10–30 min/day training should be recommended.		N/A	Patients with chronic heart or lung diseases reported the added-value of AR but suggested several improvements for a next version.
Munoz et al., 2021 [80]	Acceptability questionnaire		According to the questionnaire score, a progressive acceptance for the AR tool was observed.		*p* < 0.05 between session 2 and 4 (for female) and session 4 and 6 (for all)	Participants reached a high level of acceptance for the AR tool at the end of the experiment.
Vieira et al., 2020 [67]	Pictorial Scale		Participants provided a high to very high score with regards to the different features of the AR application.		N/A	Future designers may involve older adults using AR similarly to increase participation for users’ preferences.
CAVE						
Pedroli et al., 2018 [82]	Interview with open questions		“I felt like I was in a real park” “I was focused on the task “The environment was realistic” “I think it is easier to train with this tool” “I felt like the animals were touching me” “I felt passive and not active in the environment”		N/A	Participants were very involved in the environment and in the task. Participants forgot the context in which they were training. This may encourage patients to participate in their rehabilitation sessions.

**Table 6:** Results and author(s)’ conclusions on the feasibility of immersive technologies with a geriatric population.

Author(s), Year	Sample Size	Data Collection Method			Results	*p*-Value	Author(s)’ Conclusions
Immersive virtual reality							
Appel et al., 2020 [30]	66	Questionnaire	Quiet		Pre = 4.37 ± 1.02 Post = 4.57 ± 1.18	N/A	Did not cause side effects such as nausea, confusion, disorientation, or dizziness
			Relax		Pre = 3.9 ± 1.34 Post = 4.48 ± 1.08	N/A	
			Happy		Pre = 3.76 ± 1.53 Post = 4.27 ± 1.25	N/A	
			Adventurous		Pre = 2.79 ± 1.65 Post = 3.28 ± 1.74	N/A	
			Energetic		Pre = 2.79 ± 1.72 Post = 3.31 ± 1.67	N/A	
			Happy		Pre = 3.66 ± 1.49 Post = 3.96 ± 1.56	N/A	
			Relax		Pre = 3.39 ± 1.63 Post = 1.30 ± 0.74	N/A	
			Tense		Pre = 1.48 ± 1.11 Post = 1.34 ± 0.83	N/A	
			Upset		Pre = 1.82 ± 1.25 Post = 1.42 ± 1.12	N/A	
			Stressed		Pre = 1.94 ± 1.50 Post = 1.86 ± 1.55	N/A	
			Anxiety		Pre = 1.96 ± 1.55 Post = 1.81 ± 1.51	N/A	
Barsasella et al., 2021 [31]	60	Adverse events			0	N/A	All participants completed the study; there were no dropouts. No potential harms or symptoms were reported.
		Drop-outs			0		
Brown, 2019 [33]	10	Interview		Headset	2 people said the headset was too heavy. 1 person said their head was too small for the headset. 1 person said the headset slipped off.	N/A	The headset is suitable for most people, Precautions should be taken for people with head and neck pain. The helmet may be heavy for some users and cervical movements may create pain for those with cervical restrictions or those who are confined to a bed/chair. Vision problems may be an issue for some.
				Handheld controller	Controller in the virtual environment was not aligned in the same direction as the one in reality.	N/A	The handheld controller may be difficult to use due to non-alignment with its position in real space.
				Balance	Stability was an issue when moving, some with the sensation of head spinning or being too high. For some, feeling that there was too much movement around them in the virtual environment, as the helmet moved when actually moving. 2 participants experienced slight loss of balance.	N/A	Balance problems are possible even in people who do not have this problem. It is therefore an even greater issue for people with balance problems.
Campo-Prieto et al., 2021 [35]	4	SSQ			No symptoms	N/A	The outcomes support the feasibility of the HTC Vive.
Campo-Prieto et al., 2022 (a) [36]	32	SSQ			0 ± 0	N/A	No adverse events were reported which is important for safety.
Campo-Prieto et al., 2022 (b) [37]	12	SSQ			0 ± 0	N/A	Our findings show that a 10-week IVR protocol was feasible for nonagenarian women.
Campo-Prieto et al., 2022 (c) [38]	24	SSQ			No symptoms before and after the intervention.	N/A	The findings show that the IVR intervention is a feasible method to approach a personalized exercise program and an effective way by which to improve physical function in the target population.
Cikajlo and Peterlin Potisk, 2019 [39]	20	IMI		Q1 + Q4 (Perceived competence)	U3 [CI] = 0.8 [0.5–0.9]	*p* = 0.037	The LCD group had a slightly higher perceived competence than the VR group and had objectively less tremors, an indication of the level of pressure felt by the subjects during the experiment.
				Q2 + Q6 (Pressure/Tension)	U3 [CI] = 0.9 [0.5–1.0]	*p* = 0.422	
Crosbie et al., 2006 [40]	15	Borg Scale			5.00 ± 1.41	N/A	Similar scores between the 2 groups on the TSFQ. Stroke group had higher effort than healthy adult group → + effort required when MS deficits present. Some users in both groups had transient side effects after using VR.
		TSFQ			14.80 ± 7.73	N/A	
		Closed questionnaire on side effects (Stroke group)			1 = Yes 4 = No	N/A	
De Keersmaecker et al., 2020 [16]	28	SSQ		In park	Pre = 7.75 ± 7.40 Post = 9.08 ± 7.29	*p* > 0.05	Type of location has no effect on QSS outcome. Well tolerated regardless of where the user travels.
				In corridor	Pre = 6.95 ± 6.86 Post = 10.69 ± 12.26	*p* > 0.05	
Hoeg et al., 2021 [41]	60	SSQ			Change scores: N = 16.5 ± 13.6 O = 5.5 ± 11.3 D = 6.3 ± 9.6 Ts = 8.5 ± 8.0	N/A	The reported levels of discomfort measured with the SSQ were generally lower than anticipated.
Janeh et al., 2019 [42]	15	SSQ			Pre = 16.45 ± 16.59 Post = 15.21 ± 17.04	*p* = 0.306	Walking in VR resulted in an increase in step width, cadence, and variability of walking pattern, reflecting an insecure walking pattern during immersion in VR. Few symptoms when walking with HMD. No significant increase in symptoms.
Kim et al., 2017 [46]	22	SSQ Post RV (Healthy elderly)			6.5 ± 13.0	Difference between Parkinson’s and healthy PCs: *p* < 0.01	The higher score for people with Parkinson’s is a side effect of the medication that is present with the use of VR.
		SSQ Post RV (Parkinson)			27.5 ± 22.5		
Kruse et al., 2021 [48]	25	SSQ			Pre-intervention: 9 ±11.5 Post-intervention: 8.1 ± 11.5	*p* = 0.75	Our study showed that virtual humans or virtual content were largely accepted by the older adults.
Micarelli et al., 2019 [53]	23	SSQ	Nausea for headset + vestibular group		Pre = 2.9 ± 0.7 Post = 1.36 ± 0.5	*p* < 0.001	Reduction of adverse effects experienced after vestibular treatment with VR.
			Disorientation for headset + vestibular group		Pre = 4 ± 0.77 Post = 1.9 ± 0.7	*p* < 0.001	
		Questionnaire DHI	Headset + vestibular group		Pre = 64 ± 5.05 Post = 30.72 ± 5.67	*p* < 0.001	
			Vestibular group		Pre = 61.16 ± 7.25 Post = 33.5 ± 4.98	*p* < 0.001	
Muhla et al., 2020 [54]	21	TUG			Real = 12.84 ± 5.56 VR = 14.76 ± 8.63	*p* < 0.001	Increasing the number of steps and time to complete the TUG in virtual reality. The addition of a weight to the head (the HMD). The reduced field of view and this added weight can cause extreme rotation/reflection, which can induce stress on the musculoskeletal structures.
		Number of steps			Real = 17.16 ± 4.83 VR = 19.17 ± 6.5	*p* < 0.001	
Parijat and Lockhart, 2011 [55]	16	SSQ			Pre = 0 Post = 5.93 ± 2.46 1 day after = 0.66 ± 0.81	N/A	/
Saldana et al., 2017 [61]	13	SSQ	Pre-post test difference VR		Visit 1: −1.38 ± 2.29 Visit 2: −0.25 ± 1.91	Visit 1: *p* = 0.05 Visit 2: *p* = 0.63	No significant difference in the total SSQ, but significant differences in the Nausea subscale for the 1st visit. In addition, 1 participant did not complete the 2nd visit after experiencing symptoms of simulation-related discomfort
			Nausea subscale; Pre-post RV difference		Visit 1: −1.31 ± 1.8 Visit 2: 0.08 ± 1.83	Visit 1: *p* = 0.02 Visit 2: *p* = 0.88	
Stamm et al., 2022 (a) [62]	22	Immersive Tendency Questionnaire Presence Questionnaire			SET: 112.6 ± 12.8 ET: 104 ± 15.8	N/A	The results of the presence questionnaire total score indicated a higher perception of presence in the strength endurance training than in the endurance training exergame.
Stamm et al., 2022 (b) [63]	22	TUI Immersion			Post-intervention: 19.09	N/A	The pilot study demonstrated it would be feasible to conduct a larger RCT study using multimodal pain management in VR.
Syed-Abdul et al., 2019 [64]	30	Written Questionnaire			Perceived ease of use: 3.27 ± 0.556–3.87 ± 0.571	N/A	User experience is an important element in the ease of use and perceived usefulness of VR for older people.
Valipoor et al., 2022 [66]	29	State Trait Anxiety Inventory			Healthy older: 23.4 ± 4.6	N/A	Using a VR-based tool to manipulate features of the virtual environment and to walk through different environmental modifications is feasible for persons with Parkinson’s.
					Parkinson’s: 23.9 ± 4.7		
Augmented reality							
Bank et al., 2018 [72]	30	Questionnaire	Conviviality (for 3 groups)		69.3 ± 13.7/100	N/A	Well tolerated by patients. Patients reported an experience that was close to natural.
			Engagement (for 3 groups)		3.8 ± 0.5/7	N/A	
CAVE							
Pedroli et al., 2018 [82]	5	Questionnaire			“Motor and cognitive tasks were easy.” “The 3D glasses were not uncomfortable.” “The environment was beautiful.” “The ergo-cycle was manageable.” No nausea or discomfort related to the simulation. “It is difficult to recognize small animals” or “when they are from behind”. The sound of the bike can be confused with auditory cues. One patient was tired before the end of the task.	N/A	The system has good usability. Several patients reported difficulty in recognizing animals that were too small or not facing the subject. Some confused similar animals. Some also had difficulty discriminating auditory cues from bicycle noise. A practice session prior to using the system would familiarize the participants with the environment and address these issues.
		SUS			76.88 ± 17.00	N/A	
		Short Flow State Scale			4.33 ± 0.84	N/A	

**Table 7:** Results and author’s conclusions on the effectiveness of immersive technologies with a geriatric population.

Author(s), Year	Level of Evidence [27]	Data Collection Method		Result	*p*-Value	Author(s)’ Conclusions
Immersive virtual reality						
Barsasella et al., 2021 [31]	Randomized controlled trial	EQ-5D		Improved: Intervention: 9 (31%) Control: 1 (3.2%)	N/A	VR leads to improved quality of life, happiness, and functional fitness.
Benham et al., 2019 [32]	Quasi-experimental study	NPRS		Pre = 3.5 ± 1.73 Post = 0.9 ± 1.62	*p* = 0.002	Use of VR is significant in improving pain after 15 min of use. Provided distraction from pain. No significant effect on quality of life.
		WHOQOL-BREF		General health: Pre = 8.42 ± 1.24 Post = 8.33 ± 1.37 Physic: Pre = 14.42 ± 4.25 Post = 16.08 ± 3.90	General health: *p* = 0.66 Physic: *p* = 0.08	
Burin et al., 2021 [34]	Randomized controlled trial	Heart rate		dHRf higher in the 1PP (9.5 ± 0.6) compared to the 3PP (−1.4 ± 0.6) group.	*p* < 0.01	A significant decrease in the response time of the Stroop task after intervention was only observed in first person VR perspective (1PP).
		Stroop Task		There was no between group difference for RT.	ns	
Campo-Prieto et al., 2022 (b) [37]	Randomized controlled trial	Tinetti Test		Intervention: +10.2% improvement Control: −9.3% decrease	Between group difference: *p* = 0.032	IVR training is effective at enhancing balance and reducing the risk of falls in female nonagenarian old people’s home residents
		Timed Up and Go Test(s)		Intervention: −0.45% improvement Control: −14.8% improvement	Between group difference: *p* = 0.568	
Campo-Prieto et al., 2022 (c) [38]	Randomized controlled trial	Five Sit-to-Stand(s)		Pre-post EG: 1.75 ± 3.63 Pre-post CG: −4.38 ± 7.44	Between group difference: *p* = 0.465	IVR program has positive effects on gait, balance, and handgrip strength in institutionalized older adults, particularly.
		Tinetti		Pre-post EG: 2.84 ± 1.67 Pre-post CG: −0.81 ± 1.99	Between group difference: *p* = 0.532	
		Timed Up and Go Test(s)		Pre-post EG: −1.06 ± 4.23 Pre-post CG: −3.03 ± 4.62	Between group difference: *p* = 0.390	
		Hand Grip Strength (kg)		Pre-post EG: 4.96 ± 4.22 Pre-post CG: 1.95 ± 2.91	Between group difference: *p* = 0.691	
Cikajlo and Peterlin Potisk, 2019 [39]	Cohort study	UPDRS–Upper limb Group VR		Pre = 3.90 ± 2.26 Post = 3.30 ± 2.24	*p* = 0.2189 (between group VR and LCD)	Both technologies improved fine motor skills in the upper limb but with no significant difference between the two groups. In terms of clinical outcomes, the two were comparable.
		BBT (number of blocks) Group VR		Pre = 48.50 ± 9.37 Post = 50.10 ± 9.97	*p* = 0.285 (between group VR and LCD)	
Janeh et al., 2019 [42]	Quasi-experimental study	GAITRite	Step length–short side (cm)	Baseline = 58.34 ± 8.27 Manipulated foot = 60.45 ± 8.16	*p* > 0.05	The decrease in the visual field has no impact on the gait pattern. The manipulated foot condition with visuo-proprioceptive dissociation was the most effective method to decrease the asymmetry of the gait pattern and to adjust the step length of both legs.
			Step length–long side (cm)	Baseline = 61.34 ± 7.78 Manipulated foot = 60.80 ± 7.68	*p* > 0.05	
			Cadence (step/min)	Baseline = 102.81 ± 8.19 Manipulated foot = 97.41 ± 9.9	*p* > 0.05	
			Gait pattern asymmetry (%)	Baseline = 1.05 ± 0.04 Manipulated foot = 1.01 ± 0.06	*p* < 0.05	
			Pitch width–short side (cm)	Baseline = 10.06 ± 3.55 Manipulated foot = 12.98 ± 4.01	*p* < 0.01	
			Step width–long side (cm)	Baseline = 10.41 ± 3.54 Manipulated foot = 13.05 ± 4.02	*p* < 0.01	
Jang et al., 2020 [43]	Randomized controlled trial	Gait speed (m/s)		VR: Pre = 1.15 ± 0.33 Post = 1.19 ± 0.37 Control: Pre = 1.18 ± 0.21 Post = 1.12 ± 0.26	Between group difference: *p* = 0.02	VR-based cognitive training has a positive effect on cognition and gait in MCI patients.
		Trail Making Test		VR: Pre = 26.3 ± 7.3 Post = 24.2 ± 5.3 Control: Pre = 27.9 ± 9.2 Post = 27.8 ± 8.1	Between group difference: *p* > 0.05	
Jung et al., 2012 [44]	Randomized study with small population	TUG(s) Pre-post difference		−2.7 ± 1.9	*p* < 0.05, pre-post and between groups	Subjects on the treadmill + VR had greater improvement in balance and decrease in fall frequency than the control group. This training can be used as an effective programme for post-stroke patients with a fear of falling.
		ABC Scale (%) Pre-post difference		9.5 ± 6.0	*p* < 0.05, pre-post and between groups	
Kanyilmaz et al., 2021 [45]	Randomized controlled trial	Vertigo Symptom Scale		VR: Pre = 9 [11] Post = 4 [6.5] Control: Pre = 15 [18] Post = 11 [18]	Between group difference: *p* = 0.257	VR-based vestibular rehabilitation may benefit elderly patients with dizziness
Kim et al., 2017 [46]	Cohort study	Center of pressure displacement (CoP) (mm2)	Healthy	Mean: 168 ± 125	Pre-post: not significant for all groups Difference between parkinsonian and healthy young adults: *p* < 0.05	Greater variability in the sway zone in Parkinson’s patients → lower postural stability. Increasing results in the Mini BESTest scores showing dynamic posture improvement.
			Parkinson’s	Mean: 572 ± 1010		
		Mini BESTest	Healthy	Pre = 23 ± 4 Post = 25 ± 3	*p* > 0.05	
			Parkinson’s	Pre = 21 ± 4 Post = 23 ± 4	*p* > 0.05	
		Walking velocity (m/s)	Healthy	Pre = 1.08 ± 0.34 Post = 1.12 ± 0.27	*p* < 0.05	
			Parkinson’s	Pre = 1.16 ± 0.18 Post = 1.20 ± 0.18	*p* < 0.05	
Kiper et al., 2022 [47]	Randomized controlled trial	Geriatric Depression Scale (GDS)		VR: Pre-post = +6.3 [4.4–8.2] Control: Pre-post = +3.4 [1.5–5.3]	Between group difference: *p* < 0.001	VR therapy combined with rehabilitation is more effective at improving mood than conventional rehabilitation.
Li et al., 2020 [49]	Randomized controlled trial	Reaction time		VR Pre-post difference: *p* < 0.001 Between group difference (VR vs. control): ns		VR video games are promising at enhancing the cognition and physical health of the aging population.
		One-Leg Standing Balance Test		VR: Pre-post difference: *p* < 0.05 Between group difference: ns		
Liepa et al., 2022 [50]	Randomized controlled trial	Divided attention test		Between group difference (Immersive vs. non-immersive VR vs. control): *p* = 0.06		VR intervention has potential benefits for cognitive impairments in older adults.
		Reaction speed		Between group difference: *p* = 0.02		
		Reaction control		Between group difference: *p* = 0.03		
		Prone test		Between group difference: *p* = 0.11		
		Short Physical Performance Battery		Between group difference: *p* = 0.47		
Liu et al., 2015 [51]	Cohort study	Frequency of falls	Group VR	Test #1 = 50% (n = 6) Test #2 = 0% (n = 0)	*p* < 0.05	More pro-active and retroactive adjustments in the VR group. Decreased trunk rotation after VR training.
			Group control	Test #1 = 50% (n = 6) Test #2 = 25% (n = 2)	*p* > 0.05	
Matamala-Gomez et al., 2022 [52]	Randomized controlled trial	Fugl-Meyer Upper Extremity	Immersive VR	Between group differences: *p* < 0.00001		Immersive VR could be used to accelerate the motor functional recovery after a distal radius fracture.
			Non-immersive VR			
			Digital rehabilitation			
Micarelli et al., 2019 [53]	Cohort study	DGI scale (helmet + vestibular group)	Group VR + vestibular	Pre = 11.36 ±1.68 Post = 20 ± 1.84	N/A	Significant increase in scores on the ABC Scale and DGI which examine quality of life. More difficult to use VR headset for people with cognitive impairment. Better posture after using headset for vestibular rehabilitation.
			Group vestibular	Pre = 12.5 ± 1.62 Post = 19 ± 1.47	N/A	
		ABC scale	Group VR + vestibular	Pre = 62.54 ± 4.8 Post = 71.36 ± 4.24	N/A	
			Group vestibular	Pre = 64.91 ± 5.94 Post = 72.41 ± 6.15	N/A	
		DHI scale–total scoring	Groupe VR + vestibular	Pre = 64 ± 5.05 Post = 30.72 ± 5.67	N/A	
			Group vestibular	Pre = 61.16 ± 7.25 Post = 33.5 ± 4.98	N/A	
Parijat and Lockhart, 2011 [55]	Cohort study	Step length		Without VR = 12.20 ± 2.23 VR 5 min = 20.17 ± 9.34 VR 10 min = 18.88 ± 7.56 VR 15 min = 17.17 ± 6.34 VR 20 min = 10.31 ± 5.34 VR 25 min = 10.39 ± 3.45	Not significant between TW1 (without VR) and VR5 (after 25 min)	Decreased variation in walking parameters as the subject becomes accustomed to the task. Incoordination at the beginning of the use of virtual reality because of the different information provided by the body systems.
		Step velocity		Without VR = 5.23 ± 1.78 VR 5 min = 9.63 ± 3.55 VR 10 min = 7.19 ± 2.88 VR 15 min = 7.98 ± 1.98 VR 20 min = 6.22 ± 1.23 VR 25 min = 5.92 ± 1.91	Not significant between TW1 (without VR) and VR5 (after 25 min)	
Parijat et al., 2015 [56]	Cohort study	Joint Amplitude (JA) Plantar Flexion (PF)	VR	Initial = 104.60 ± 6.22 Final = 105.38 ± 4.26	*p* > 0.05	The increase in joint amplitude is attributable to more rapid muscle activation.
			Control	Initial = 110.32 ± 4.55 Final = 108.87 ± 6.78	*p* > 0.05	
		JA Knee flexion	VR	Initial = 30.23 ± 8.45 Final = 23.04 ± 8.68	*p* > 0.05	
			Control	Initial = 24.59 ± 5.39 Final = 21.24 ± 4.38	*p* > 0.05	
		JA hip flexion	VR	Initial = 15.44 ± 6.96 Final = 12.61 ± 5.45	*p* > 0.05	
			Control	Initial = 18.70 ± 3.47 Final = 16.42 ± 2.53	*p* > 0.05	
		JA trunk extension	VR	Initial = 35.44 ± 13.96 Final = 28.61 ± 10.45	*p* > 0.05	
			Control	Initial = 38.70 ± 13.47 Final = 39.42 ± 12.53	*p* > 0.05	
		Muscle activation MG (ms)	VR	Initial = 178 ± 35.67 Final = 180 ± 12.67	*p* > 0.05	
			Control	Initial = 189 ± 24.29 Final = 179 ± 25.29	*p* > 0.05	
		Muscle activation TA (ms)	VR	Initial = 187 ± 28.26 Final = 180 ± 11.69	*p* > 0.05	
			Control	Initial = 188 ± 21.23 Final = 178 ± 12.69	*p* > 0.05	
		Muscle Activation MHs (ms)	VR	Initial = 159 ± 14.76 Final = 138 ± 11.37	*p* < 0.05	
			Control	Initial = 168 ± 15.28 Final = 156 ± 13.39	*p* < 0.05	
		Muscle activation VL (ms)	VR	Initial = 239 ± 33.54 Final = 222 ± 14.54	*p* > 0.05	
			Control	Initial = 245 ± 25.76 Final = 255 ± 15.99	*p* > 0.05	
Phu et al., 2019 [57]	Non-randomized prospective study (quasi-experimental)	Grip force (% pre-post change) (CI_95%_)		EX: 11.32 (5.84, 17.08) BRU: 6.82 (0.77, 13.24) Control: −0.07 (−5.61, 5.79)	EX: <0.001 BRU: 0.027 Control: 0.98	BRU is effective at improving static and dynamic balance and physical performance of older people in community settings. Decreased fear of falling by at least 10% in EX and BRU groups. BRU had similar physical increases to EX group but with half the training time (**possible ceiling effect in EX group). No obvious differences between BRU and EX → BRU could be equally effective at improving physical performance and fall risk in older adults. Significant improvements in 5TSTS, TUG, FSST, walking speed, FES-I, and grip strength in EX and BRU groups vs. control group.
		FTSTS (% pre-post change) (CI_95%_)		EX: −29.84 (−35.23, −23.99) BRU: −26.69 (−33.22, −19.52) Control: −21.79 (−30.00, −12.62)	EX: <0.001 BRU: <0.001 Control: <0.001	
		TUG (%pre-post change) (CI_95%_)		EX: −20.33 (−24.99, −15.38) BRU: −23.30 (−28.42, −17.83) Control: −4.31 (−10.68, 2.50)	EX: <0.001 BRU: <0.001 Control: 0.209	
		FSST (%pre-post change) (CI_95%_)		EX: −23.95 (−30.45, −16.83) BRU: −18.87 (−26.89, −9.96) Control: −16.72 (−24.45, −8.20)	EX: <0.001 BRU: <0.001 Control: <0.001	
		Walking velocity (%pre-post change) (CI_95%_)		EX: 0.15 (0.10, 0.20) BRU: 0.12 (0.07, 0.17) Control: 0.06 (0.02, 0.10)	EX: <0.001 BRU: <0.001 Control: 0.007	
		Falls Efficacy Scale-International (%pre-post change) (CI_95%_)		EX: −15.7 (−21.6, −9.5) BRU: −11.3 (−18.2, −3.8) Control: −1.6 (−8.8, 6.1)	EX: <0.001 BRU: 0.004 Control: 0.676	
Rebelo et al., 2021 [58]	Randomized controlled trial	Dynamic Gait Index		Immersive VR: Pre-post difference = 3 (95% CI: 1.4–4.6) Control: Pre-post difference = 3.9 (95% CI: 2.2–5.6)	Effect size (between group difference): 0.88 (95% CI: −1.35–3.12)	Virtual Reality training proved to be effective for balance-related outcomes, although not superior to conventional therapy.
Rutkowski et al., 2021 [59]	Randomized controlled trial	Hospital Anxiety Depression Scale		Immersive VR: Pre = 18.3 ± 4.9 Post = 13.2 ± 4 Control: Pre = 15.2 ± 4.5 Post = 15.7 ± 5.3	Immersive VR: Pre-post difference: *p* < 0.001 Control group: *p* = 0.612	Immersive VR decreases depression and anxiety.
Sakhare et al., 2021 [60]	Cohort study	Montreal Cognitive Assessment		Pre = 26 ± 2.7 Post = 25.8 ± 3.7	Effect size = 0.06 *p* > 0.05	Immersive VR + exercises leads to changes in brain volume, memory, and executive functions.
		Gray Matter Volume		Pre = 633 ± 32.7 Post = 637.6 ± 25.6	Effect size = 0.38 *p* < 0.05	
Stamm et al., 2022 (b) [60]	Randomized controlled trial	Pain intensity (numeric rating scale)		Immersive VR: Pre = 3.6 ± 2.4 Post = 2.9 ± 2 Control: Pre = 2.9 ± 2.4 Post = 1.6 ± 1.5	Immersive VR: Pre-post difference: *p* = 0.535 Control: Pre-post difference: *p* = 0.07	A pain intensity reduction can be achieved with immersive VR, although not significantly more than with multimodal pain therapy.
Szczepanska-Gieracha et al., 2021 [65]	Randomized controlled trial	Geriatric Depression Scale		Immersive VR: Pre = 12.3 ± 4.5 Post = 7.3 ± 2.6 Control: Pre = 12.3 ± 4.5 Post = 11.8 ± 2.6	Immersive VR: Pre-post difference: *p* < 0.001 Control: Pre-post difference *p* = 0.61	Immersive VR decreases the intensity of depressive symptoms stress and anxiety levels in older women
Yalfani et al., 2022 [68]	Randomized controlled trial	Pain (Visual Analogic Scale)		Between group difference: Effect size = 0.84 *p* = 0.001		Immersive VR can reduce older adults’ symptoms and enhance their quality of life.
		Fall Risk Index		Between group difference: Effect size = 0.45 *p* = 0.001		
		Physical Health		Between group difference: Effect size = 0.58 *p* = 0.001		
		Mental Health		Between group difference: Effect size = 0.41 *p* = 0.001		
		Quality of Life		Between group difference: Effect size = 0.59 *p* = 0.001		
Yang et al., 2022 [69]	Randomized controlled trial	Mini Mental Scale Exam		Immersive VR: Pre = 27.2 ± 1.9 Post = 28.1 ± 1.7 Exercise: Pre = 26.9 ± 1.7 Post = 27.8 ± 1.6	Pre-post difference: Immersive VR: *p* < 0.05 Exercise: *p* < 0.05	Immersive VR and exercise training enhances brain, cognitive, and physical health in older adults with MCI
		EEG band power: theta		Between group comparison (Immersive VR vs. Exercise): *p* = 0.036		
Yoon et al., 2020 [70]	Randomized controlled trial	Timed Up and Go Test		Immersive VR: Pre = 34.1 ± 3.4 Post = 19 ± 5.7 Control: Pre = 36.2 ± 3.7 Post = 21.4 ± 5.8	Pre-post difference: Immersive VR: *p* < 0.001 Control: *p* < 0.001	VR training produced better early balance ability and knee function than passive motion and exercise therapy.
Zak et al., 2022 [71]	Randomized controlled trial	Single-Leg Stand Open Eyes		Immersive VR: Pre = 14.4 ± 4.2 Post = 16.4 ± 2.7	Pre-post difference: *p* < 0.001	Immersive VR application enhances static balance.
Augmented reality						
Bank et al., 2018 [72]	Cohort study	Measurement with VR	Hand opening adjustment	Initial opening > opening during interaction	N/A	Smaller cubes are more difficult to handle. The presence of obstacles makes the movement path longer and the speed of execution slower.
			Balloon reach on screen	Healthy = 98.0 ± 2.9 Parkinson’s = 96.8 ± 2.9 Stroke = 95.5 ± 2.9	*p* > 0.05	
Chen et al., 2020 [74]	Randomized controlled trial	Berg Balance Scale		Intervention: Pre = 50 ± 2.1 Post = 54 ± 1.1 Control: Pre = 49.2 ± 4.5 Post = 51.1 ± 4.7	Between group difference: *p* = 0.044	A VR-augmented training system can achieve training goals more readily than traditional Tai Chi.
		Timed Up and Go Test(s)		Intervention: Pre = 8.7 ± 0.7 Post = 6.9 ± 0.9 Control: Pre = 9 ± 1.8 Post = 8.4 ± 1.6	Between group difference: *p* = 0.015	
Ferreira et al., 2022 [75]	Cohort study	Trail Making Test		AR: 42.1 ± 17.1 Cycle: 46.4 ± 29.5 Control: 39.2 ± 17.9	Between group difference: *p* = 0.226	The AR session showed no significant improvements compared with the session with the cycle ergometer and without exercise in verbal fluency, reaction time, and cognitive flexibility.
Fischer et al., 2007 [76]	Randomized study with small population	WMFT Time(s) Pneumatic Orthosis Group		Pre = 92 ± 36.4 Post = 79.1 ± 34.2 Follow-up = 76.1 ± 37.2	*p* = 0.02 (total population)	Small significant increase in task performance on the WMFT No significant change in biomechanical measures of the hand AR allowed faster transitions between tasks and more opportunities to practice grasping objects that are not available in a conventional practice environment The limited field of view (28°) was a problem for some subjects as it was difficult to see the object and move the arm independently of the neck It is feasible to incorporate mechatronic devices and VR into hand rehabilitation, even for individuals with severe stroke. The effectiveness of these tools has yet to be demonstrated in a severely impaired population. Participants were generally enthusiastic about the addition of VR to training.
		BBT (number of blocks) Pneumatic Orthosis Group		Pre = 4 ± 7.1 Post = 3 ± 6.6 Follow-up = 4 ± 8.3	*p* = 0.09 (total population)	
		Fugl-Meyer Total Score Pneumatic Orthosis Group		Pre = 19 ± 9 Post = 18 ± 10 Follow-up = 20 ± 11	*p* = 0.08 (total population)	
		RLA Time (seconds) Pneumatic Orthosis Group		Pre = 65.7 ± 15.5 Post = 58.9 ± 37.7 Follow-up = 63.7 ± 46.8	Ø significative (total population)	
		Standardized Grip Force Pneumatic Orthosis Group		Pre = 0.21 ± 0.1 Post = 0.20 ± 0.1 Follow-up = 0.25 ± 0.1	*p* > 0.20 (total population)	
		Spasticity Pneumatic Orthosis Group		Pre = 1.2 ± 0.8 Post = 1.3 ± 1.0 Follow-up = 1.7 ± 1.3	*p* > 0.20 (total population)	
		Isometric Flexion (N-m) Pneumatic Orthosis Group		Pre = 2.5 ± 1.6 Post = 2.8 ± 1.5 Follow-up = 2.9 ± 1.5	*p* > 0.20 (total population)	
		Isometric Extension (N-m) Pneumatic Orthosis Group		Pre = 0.3 ± 0.6 Post = 0.3 ± 0.6 Follow-up = 0.3 ± 0.6	*p* > 0.20 (total population)	
		ROM Extension (°) Pneumatic Orthosis Group		Pre = 10.9 ± 15.5 Post = 9.8 ± 14.9 Follow-up = 12.1 ± 11.6	*p* > 0.20 (total population)	
Jeon et al., 2020 [77]	Randomized controlled trial	Appendicular Skeletal Muscle Mass (kg)		AR: Pre = 15.3 ± 1.8 Post = 15.8 ± 1.7 Control: Pre = 15.7 ± 1.6 Post = 15.1 ± 1.4	Between group difference: *p* = 0.003	AR-based exercise program is effective at inducing physical activity in the elderly.
Koroleva et al., 2020 [78]	Randomized controlled trial	Fugl-Meyer Upper Extremity		AR + CT: Pre = 35 [31–40] Post = 61 [56–64] AR alone: Pre = 39 [28–45] Post = 63 [58–64] Control: Pre = 39 [15–45] Post = 54 [47–59]	N/A	AR rehabilitation improves the post-stroke clinical condition.
		Fugl-Meyer Lower Extremity		AR + CT: Pre = 24 [21–27] Post = 33 [29–34] AR alone: Pre = 26 [22–28] Post = 33 [29–34] Control: Pre = 24 [20–29] Post = 29 [27–33]	N/A	
		BDNF Level		AR + CT: Pre-post = −525 [−1073–698] AR alone: Pre-post = −1231 [−1178–2120] Control: Pre-post = −2415 [−3117–760]	AR + CT vs. Control: *p* = 0.049 AR alone vs. Control: *p* = 0.021	
Koroleva et al., 2021 [79]	Randomized controlled trial	Fugl-Meyer Upper Extremity		Early rehabilitation + AR: Pre = 42 [38–50] Post = 61 [56–64]	Pre-post difference: *p* < 0.001	AR training is effective as a separate rehabilitation method in the early recovery period of moderately severe, hemiparalytic, and ischemic stroke.
Munoz et al., 2021 [80]	Cohort study	Shoulder Abduction (angle)		Pre-post differences: *p* < 0.001		AR leads to improved physical achievements.
		Double Leg Squat (angle)				
Yoo et al., 2013 [81]	Non-randomized prospective study	Berg Balance Scale (Training VR)		Pre = 47.60 ± 5.36 Post = 53.50 ± 2.30	*p* < 0.001	Improvement of the hip and ankle strategies that allow to maintain balance during unconscious body movements (Static movement = Ankle strategy; Dynamic movement = Hip strategy).
		Falls Efficacy (Training VR)		Pre = 14.50 ± 4.58 Post = 11.80 ± 3.71	*p* < 0.05	
		Walking Velocity (cm/s) (Training VR)		Pre = 79.83 ± 13.22 Post = 99.18 ± 11.56	*p* < 0.01	
		Cadence (step/min) (Training VR)		Pre = 100.79 ± 9.92 Post = 116.73 ± 8.81	*p* < 0.001	
CAVE						
No study						

**Figure A1:**
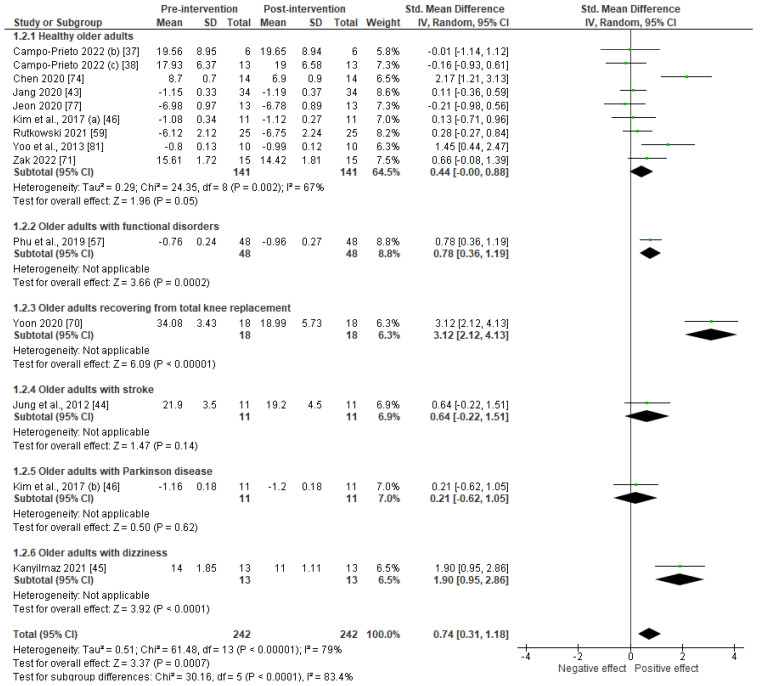
Forest-plot of effects of interventions using immersive technologies on gait speed—subgroup analyses to observe the influence of pathology.

## Data Availability

No new data were created in this review study. Data sharing is not applicable to this article.

## Appendix A

**Table A1:** Search strategy in each database.

MEDLINE (PubMed)	
S1	aged[MeSH Terms]
S2	Elderly[Title/Abstract] OR Aged[Title/Abstract] OR Older[Title/Abstract] OR Elder[Title/Abstract] OR Geriatric*[Title/Abstract]
S3	S1 OR S2
S4	Virtual reality[MeSH Terms]
S5	(Immersive[Title/Abstract] AND technolog*[Title/Abstract]) AND (“virtual realit*”[Title/Abstract]) OR VR[Title/Abstract] OR “Augmented realit*”[Title/Abstract] OR “HTC VIVE”[Title/Abstract] OR Oculus[Title/Abstract] OR “simulated environment*”[Title/Abstract] OR “artificial environment*”[Title/Abstract] OR “computer* simulat*”[Title/Abstract]
S6	S4 OR S5
S7	(Sports[MeSH Terms]) AND (Exercise[MeSH Terms])
S8	“Physical activit*”[Title/Abstract] OR exercice*[Title/Abstract] OR sport*[Title/Abstract]
S9	S7 OR S8
S10	S3 AND S6 AND S9
CINAHL Plus with Full Text (EBSCOhost)	
S1	(MH « Aged+ »)
S2	TI(Elderly OR Aged OR Older OR Elder OR Geriatric*) OR AB (Elderly OR Aged OR Older OR Elder OR Geriatric*)
S3	S1 OR S2
S4	(MH “Virtual Reality”) OR (MH “Augmented Reality”)
S5	TI((Immersive AND technolog*) OR “virtual realit*” OR VR OR “Augmented realit*” OR “HTC VIVE” OR Oculus OR “simulated environment*” OR “artificial environment*” or “computer* simulat*”) OR AB ((Immersive AND technolog*) OR “virtual realit*” OR VR OR “Augmented realit*” OR “HTC VIVE” OR Oculus OR “simulated environment*” OR “artificial environment*” or “computer* simulat*”)
S6	S4 OR S5
S7	(MH “Physical Activity”) OR (MH “Sports+”) OR (MH “Exercise+”)
S8	TI(« Physical activit* » OR exercice* OR sport*) OR AB (« Physical activit* » OR exercice* OR sport*)
S9	S7 OR S8
S10	S3 AND S6 AND S9
Embase	
S1	‘aged’/exp OR ‘aged’:ti,ab,kw OR ‘aged patient’:ti,ab,kw OR ‘aged people’:ti,ab,kw OR ‘aged person’:ti,ab,kw OR ‘aged subject’:ti,ab,kw OR ‘elderly’:ti,ab,kw OR ‘elderly patient’:ti,ab,kw OR ‘elderly people’:ti,ab,kw OR ‘elderly person’:ti,ab,kw OR ‘elderly subject’:ti,ab,kw OR ‘senior citizen’:ti,ab,kw OR ‘geriatric’/exp OR geriatric
S2	‘virtual reality’/exp OR ‘virtual reality’:ti,ab,kw OR ‘augmented reality’/exp OR ‘augmented reality’:ti,ab,kw OR ‘virtual reality system’/exp OR ‘vr interface’:ti,ab,kw OR ‘vr system (virtual reality)’:ti,ab,kw OR ‘virtual reality interface’:ti,ab,kw OR ‘virtual reality system’:ti,ab,kw OR ‘htc vive’/exp OR ‘htc vive’ OR oculus:ti,ab OR ‘artificial environment’:ti,ab OR ‘simulated environment’:ti,ab OR ‘computer simulation’/exp OR ‘computer simulation’:ti,ab,kw OR ‘computer-based simulation’:ti,ab,kw
S3	‘sport’/exp OR ‘sport’:ti,ab,kw OR ‘sports’:ti,ab,kw OR ‘exercise’/exp OR ‘effort’:ti,ab,kw OR ‘exercise’:ti,ab,kw OR ‘exercise performance’:ti,ab,kw OR ‘exercise training’:ti,ab,kw OR ‘fitness training’:ti,ab,kw OR ‘fitness workout’:ti,ab,kw OR ‘physical conditioning, human’:ti,ab,kw OR ‘physical effort’:ti,ab,kw OR ‘physical exercise’:ti,ab,kw OR ‘physical work-out’:ti,ab,kw OR ‘physical workout’:ti,ab,kw OR ‘physical activity’/exp OR ‘activity, physical’:ti,ab,kw OR ‘physical activity’:ti,ab,kw
S4	S1 AND S2 AND S3
Scopus	
S1	(TITLE-ABS-KEY (“aged”) OR TITLE-ABS-KEY (“elder*”) OR TITLE-ABS-KEY (“older”) OR TITLE-ABS-KEY (“geriat*”))
S2	(TITLE-ABS-KEY (“virtual reality”) OR TITLE-ABS-KEY (“VR”) OR TITLE-ABS-KEY (“computer simulation”) OR TITLE-ABS-KEY (“Oculus”) OR TITLE-ABS-KEY (“htc vive”) OR TITLE-ABS-KEY (“augmented reality”))
S3	(TITLE-ABS-KEY (“sport*”) OR TITLE-ABS-KEY (“physical activity”) OR TITLE-ABS-KEY (“exercise*”)
S4	S1 AND S2 AND S3
